# Transfer learning based deep architecture for lung cancer classification using CT image with pattern and entropy based feature set

**DOI:** 10.1038/s41598-025-13755-0

**Published:** 2025-08-02

**Authors:** Nithya R, Vidhyapathi C.M

**Affiliations:** School of Electronics Engineering, Vellore Institute of Technology, Vellore, Tamil Nadu 632014 India

**Keywords:** Adaptive Gaussian filtering, Improved Attention-based ResU-Net, Improved entropy, Improved LeNet with Transfer Learning, Cancer imaging, Cancer prevention, Lung cancer, Diagnosis, Medical imaging

## Abstract

Early detection of lung cancer, which remains one of the leading causes of death worldwide, is important for improved prognosis, and CT scanning is an important diagnostic modality. Lung cancer classification according to CT scan is challenging since the disease is characterized by very variable features. A hybrid deep architecture, ILN-TL-DM, is presented in this paper for precise classification of lung cancer from CT scan images. Initially, an Adaptive Gaussian filtering method is applied during pre-processing to eliminate noise and enhance the quality of the CT image. This is followed by an Improved Attention-based ResU-Net (P-ResU-Net) model being utilized during the segmentation process to accurately isolate the lung and tumor areas from the remaining image. During the process of feature extraction, various features are derived from the segmented images, such as Local Gabor Transitional Pattern (LGTrP), Pyramid of Histograms of Oriented Gradients (PHOG), deep features and improved entropy-based features, all intended to improve the representation of the tumor areas. Finally, classification exploits a hybrid deep learning architecture integrating an improved LeNet structure with Transfer Learning (ILN-TL) and a DeepMaxout (DM) structure. Both model outputs are finally merged with the help of a soft voting strategy, which results in the final classification result that separates cancerous and non-cancerous tissues. The strategy greatly enhances lung cancer detection’s accuracy and strength, showcasing how combining sophisticated neural network structures with feature engineering and ensemble methods could be used to achieve better medical image classification. The ILN-TL-DM model consistently outperforms the conventional methods with greater accuracy (0.962), specificity (0.955) and NPV (0.964).

## Introduction

Lung cancer remains one of the most prevalent and deadly forms of cancer worldwide, with significant health implications, particularly in countries like Italy, where it ranks third in prevalence according to the World Health Organization (WHO)^1,2^. It is primarily classified into two types: Small Cell Lung Cancer (SCLC) and Non-Small Cell Lung Cancer (NSCLC), with the latter accounting for 80–85% of all cases and contributing to approximately 1.6 million deaths annually^3^. Accurate and early diagnosis of lung cancer is crucial, yet it often goes undetected in the early stages due to the long latency period before symptoms appear^4^.

Recent advances in imaging technologies, particularly Computed Tomography (CT), have revolutionized the early detection of lung cancer^5,6^. CT scans, particularly low-dose CT (LDCT), have proven effective in detecting lung cancer at an early stage, thus reducing mortality rates^7^. However, the challenge remains in interpreting CT images due to the presence of indeterminate nodules, which require follow-up scans for better assessment of malignancy^8^. The reliance on manual interpretation by radiologists can lead to inaccuracies, especially when distinguishing between benign and malignant nodules^9^.

To overcome these limitations, machine learning (ML) and deep learning (DL) techniques have emerged as powerful tools in the analysis of medical imaging data^10^. By automatically detecting relevant features in CT scans, DL models, specifically CNNs, have indicated the possibility of improving the accuracy of lung cancer diagnosis and aiding radiologists in decision-making. Various architectures, such as VGG16, have been explored for lung cancer classification, with promising results in differentiating between malignant and benign lesions^11^.

Furthermore, artificial intelligence (AI)-based approaches, including the fusion of radiomic features with DL models, have gained attention for their ability to enhance diagnostic precision by capturing subtle image characteristics^12^. Radiomics involves the extraction and quantification of texture-based features from medical images, which can be used in conjunction with AI algorithms to improve prediction accuracy for various cancer subtypes, including adenocarcinoma and squamous cell carcinoma^13^.

While AI methods have proven effective, challenges still exist, such as the need for large, annotated datasets and the integration of multimodal imaging data^14^. Additionally, ethical concerns surrounding AI include the confidentiality and privacy of patient data, which are vital in medical settings. Allowing model training to take place locally on individual devices ensures that sensitive patient information is kept secure and protected from potential security breaches^43^. Moreover, the risk of over-reliance on automated systems remains a significant point of discussion^15^. Despite these challenges, AI continues to push the boundaries of lung cancer detection, offering the potential for faster, more accurate, and more personalized diagnostic tools that could ultimately improve patient outcomes and survival rates^16,17^. This paper introduces a novel transfer learning-based deep learning framework for lung cancer classification on CT images. The main contribution of this paper is:

- Introducing an improved Attention-based ResU-Net model to segment preprocessed CT images. Here, introduce a Residual Dual Channel Attention Block, which facilitates the indication of important features. This block allows the network to adaptively change the channel-wise feature importance according to the input, leading to better generalization and better performance on different datasets.
- Contributing an improved entropy-based feature extraction technique. This method introduces the dynamic modulation of the effects of both Shannon entropy and normalized entropy, based on the local features of the segmented image. This modulation increases the model’s capacity to retain spatial distribution information, which is vital in correctly discriminating between the various regions within the segmented images.
- Proposing an improved LeNet integrated with a transfer learning (ILN-TL) framework for lung cancer classification. The improved LeNet architecture includes additional convolution layers and Gaussian normalization layers to enhance the model to better train complex features. The suggested model is also integrated with the DeepMaxout model, and both are combined using soft voting, which improves classification accuracy and offers a stable framework for accurate lung cancer detection.

The remaining part of this paper is arranged as follows: Section "Literature review" gives a review of previous lung cancer classification models. Section "Proposing a hybrid DL model for lung cancer classification using ILN-TL-DM" outlines the process of the proposed hybrid deep model, together with a brief overview of the methodology for lung cancer classification. Section 4 gives the investigational outcomes and discussion of the proposed method. Lastly, Section "Conclusion" presents a conclusion of the paper, presenting a conclusion of the main findings and contributions.

## Literature review

In 2024, Jia Uddin^18^ has proposed a DL based technique for early lung cancer detection from chest CT scans to obtain high diagnostic accuracy even from a relatively small dataset. They trained an ensemble of CNNs with segmentation and data augmentation procedures to improve model performance. The model proposed performed remarkably well, with high accuracy in separating cancerous from non-cancerous scans and classifying normal, benign, or malignant nodules. These results show the potential of the model to aid radiologists by minimizing diagnostic errors at the initial stages of lung cancer.

In 2024, Lavina Jean Crasta et al.^19^ has suggested a new DL model named ATT-DenseNet for lung cancer diagnosis from CT and histopathological images. Through the improvement of the baseline DenseNet architecture by incorporating an attention mechanism, the model can selectively pay attention to key areas in medical images, resulting in enhanced feature extraction and diagnostic performance. This is done by eliminating redundant model parameters while enhancing knowledge of the intricate structure in cancer images. The model performed better compared to conventional networks such as AlexNet and SqueezeNet and had an extremely high accuracy on CT scans.

In 2024, Sampangi Rama Reddy BR et al.^20^ has introduced a DL framework integrating 3D-VNet for segmentation and 3D-ResNet for classification to identify and diagnose lung cancer from CT scans. Targeting challenges such as small annotated datasets, class imbalance, and high similarity between benign and malignant nodules, the model leverages 3D representations of CT scans for increased spatial context. Segmentation model reached a high Dice Similarity Coefficient, and classification model reached high accuracy, sensitivity and specificity. This two-architecture model strongly surpassed other models in detection accuracy and false-positive reduction.

In 2024, Anindita Saha et al.^21^ has proposed a new SNN structure for early classification and detection of lung cancer based on CT scan images. This method combines several NN models to enhance the precision of categorization by leveraging their strengths through feature extraction and transfer learning. Initially, lung areas are segmented by image processing, and then features are extracted from the segmented nodules. These characteristics are subsequently classified with the SNN model, which attained a high classification accuracy, performing better than standard single-network models. The research proves the potential of the SNN model for clinical use and proposes future enhancement through the use of swarm intelligence-based optimization methods to further improve model performance.

In 2024, M. Mohamed Musthafa et al.^22^ has proposed an ML-based method with a CNN for diagnosing different stages of lung cancer based on CT scans, with the view to enhancing early detection precision and minimizing invasive interventions. The model was thoroughly preprocessed (resizing, normalization, Gaussian blurring) and employed the SMOTE strategy to compensate for class imbalance to improve the classification of poorly represented stages of cancer. The model reached an impressive high positive metrics for every class. The approach has a lot of potential to help clinicians make wise and quick diagnostic choices, which could ultimately result in early interventions and better patient outcomes.

In 2024, C. Venkatesh et al.^23^ has proposed a new DL-based method for accurate and efficient detection of lung cancer from CT scan images. To counter limitations in current machine learning systems, which have lower accuracy and high computation time, the research utilizes CNNs in combination with pre-processing methods such as median filtering as well as patch processing to improve image quality. The CNN receives segmented CT images to extract features and classify them. The approach was designed to improve diagnostic time while ensuring increased accuracy through low- and high-level feature analysis.

In 2024, S. K. B. Sangeetha et al.^24^ has suggested a new MFDNN to improve the reliability and accuracy of lung cancer diagnosis by fusing different sources of data, including medical imaging, genomics, and clinical data. The DL structure features integration of different diagnostic modalities in overcoming the limitations of single-source analysis and makes use of electronic health records to make more accurate decisions. By recording a remarkable accuracy coupled with robust precision, recall, and F1-score values, MFDNN proves its dominance over traditional models such as ResNet, DNN, and CNN. The research also stresses the need for ethical deployment of AI, pointing to the necessity of rigorous validation and regulatory guidelines prior to clinical implementation. In total, the method represents a significant breakthrough in AI-based cancer diagnosis, providing a strong and comprehensive solution to early and precise lung cancer detection.

In 2025, Muna Alsallal et al.^25^ has proposed a hybrid model that unifies radiomic features with attention mechanisms and DL to enhance the classification performance of lung cancer subtypes based on CT scans. By combining conventional radiomics with a DeepCNN augmented by attention modules, the model could selectively attend to diagnostically important areas within tumor-bearing slices. The research utilized CT scans from five healthcare facilities and diagnosed several subtypes of lung cancer. Feature selection methods such as NMF and RFE, and ensemble classifiers such as XGBoost and Stacking, assisted in enhancing prediction performance and model stability. The model was highly accurate, AUC and sensitivity, which indicates its capacity to support clinical decision-making and future incorporation with multimodal imaging devices.

In 2024, Yucheng Liu et al.^26^ has suggested a DL-based pipeline for lung nodule malignancy classification and pulmonary fibrosis estimation from chest CT scans. The authors created a 3D classification model by combining an auto-segmentation model as well as an 3D AG-Net. The model was trained with in-house CT datasets and had three forms: nodule alone, nodule with nearby microenvironment, and nodule with semantic fibrosis metadata. The outcomes showed that incorporating the fibrotic microenvironment enhanced the sensitivity, accuracy and AUC of the model, with optimal performance when including semantic fibrosis information.

In 2024, Nasr Y. Gharaibeh et al.^42^ has presented a lung cancer diagnostic technique based on artificial intelligence (AI). Images obtained from the LUNA 16 lung cancer dataset were preprocessed using the Butterworth smooth filter technique. The bi-level feature selection step follows, where features like diameter, margin, spiculation, lobulation, subtlety, and malignancy were selected utilizing the Chaotic Crow Search Algorithm and Random Forest (CCSA-RF) technique. The Multi-space Image Reconstruction (MIR) approach with Grey Level Co-occurrence Matrix (GLCM) was then used to extract features. Lastly, the Sparse Convolutional Neural Network (SCNN) technique with a Probabilistic Neural Network (PNN) was used to develop the Lung Tumor Severity Classification (LTSC). By employing the PNN algorithm, which lowers complexity and effectively produces classification results, the created approach can identify benign, normal, and malignant lung cancer images. However, a number of factors affect the method’s effectiveness, such as the quality and resolution of the images, and these could affect the model’s performance.

Table 1 shows the literature review part summarizes the earlier work on lung cancer classification, comparing methodologies, features, and challenges.

**Table 1:** Merits and Disadvantages of Conventional Works.

Researchers [References]	Methods	Merits	Disadvantages
Jia Uddin^18^	CNN	It performs both binary and multi-class classification, making it versatile for clinical use	The method might not perform equally well across different imaging modalities or scanner settings
Lavina Jean Crasta et al.^19^	Modified DenseNet	The attention mechanism ensures focus on tumor-relevant zones, improving diagnostic precision	The model may require high computational resources during training, limiting its use in low-resource settings
Sampangi Rama Reddy BR et al.^20^	3D-VNet and 3D-ResNet	High-resolution segmentation ensures precise localization of nodules regardless of size and shape	Training 3D models demands substantial computational resources and longer processing times
Anindita Saha et al.^21^	SNN	The modular design enables flexible tuning and upgrades in different stages of processing	Interpretability of the model’s decisions is not discussed, limiting clinical transparency
Mohamed Musthafa et al.^22^	CNN	It is trained on a preprocessed dataset with balanced class distributions, improving reliability across all lung cancer stages	It lacks interpretability, making it difficult for clinicians to understand the decision-making method
C. Venkatesh et al.^23^	CNN	The model is utilized to automatically extract deep features from segmented CT images, enabling robust classification of cancer types	The clustering segmentation approach may not perform well on complex or overlapping nodules with irregular shapes, reducing segmentation accuracy
S. K. B. Sangeetha et al.^24^	MFDNN	Genomic data integration allows the model to consider molecular and genetic markers of cancer progression, enhancing diagnostic depth	The complexity of multimodal integration requires substantial computational resources and well-labeled datasets
Muna Alsallal et al.^25^	DeepCNN	The inclusion of attention mechanisms improves feature interpretability and focuses the model on diagnostically important regions	Manual selection of tumor slices from CT scans could introduce bias and limits full automation
Yucheng Liu et al.^26^	3D U-Net, 3D AG-Net	Attention maps and CAM were employed to qualitatively analyze and visualize the decision-making method of the model	The computational complexity of 3D DL models might be a drawback in resource-scarce settings
Nasr Y. Gharaibeh et al.^42^	SCNN-PNN	This method reduces computational complexity and enhances the classification accuracy	The model’s performance relies on the resolution of the image

### Problem statement

Even with the advances in the classification of lung cancer via DL methods, a number of traditional methods continue to suffer from important limitations that limit their practicality in clinical environments. For example, CNN-based methods^18,23^ tend to find it difficult to extract hierarchical features from CT scans because of their comparatively shallow architecture, which results in suboptimal performance when handling complicated patterns in the images. In addition, conventional models tend to be disadvantaged by weak generalization when trained on small data, as they do not take advantage of the strong feature hierarchies learned from large-scale pre-trained models. Another critical problem is that these models have a weak capability to segment and classify nodules in CT scans, especially in scenarios where there is no sufficiently annotated large dataset or in the case of scans with different qualities. Moreover, models such as DenseNet^19^ and ResNet^20^, while being strong, tend to consume a lot of computational resources during training, which makes them less scalable in resource-limited settings. To address these shortcomings, the method proposed in this paper seeks to improve feature extraction, enhance segmentation accuracy, and utilize transfer learning, thus enhancing classification performance.

## Proposing a hybrid DL model for lung cancer classification using ILN-TL-DM

**Fig. 1:**
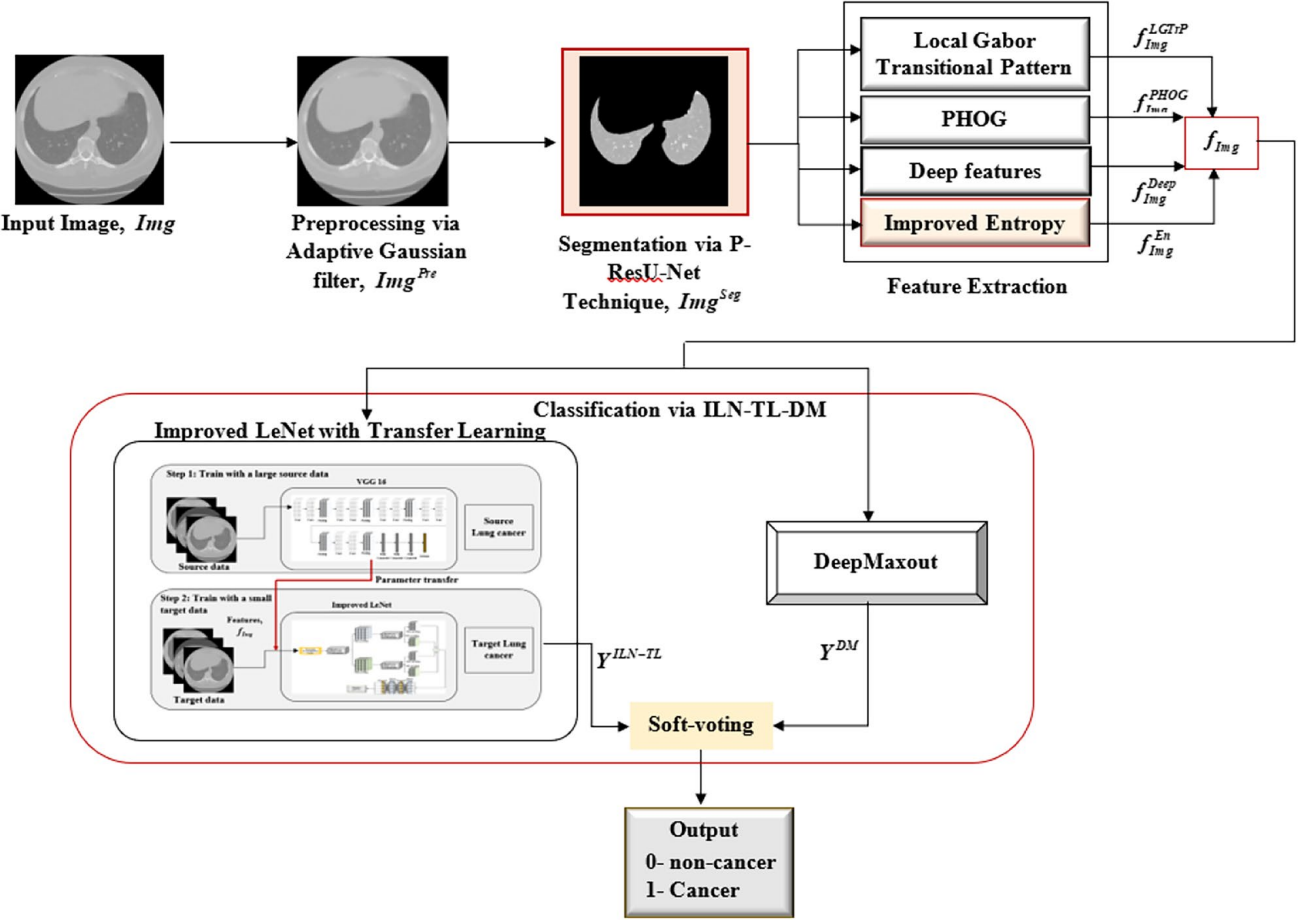
Proposed a hybrid deep learning model for lung cancer classification.

This work proposed a novel hybrid DL model for lung cancer classification based on CT images. The entire approach is outlined below:

- Firstly, Adaptive Gaussian filtering is employed to the input CT images to reduce noise and improve the image quality.
- Secondly, the preprocessed image is segmented through an improved Attention-based ResU-Net (P-ResU-Net) model. This model proposes a Residual Dual Channel Attention Block, which learns to adaptively modify channel-wise feature importance, enhancing the generalization and performance of the model over various datasets.
- Subsequently, the segmented image is extracted from which the major features are obtained. The features are Local Gabour Transitional Pattern (LGTrP), PHOG (Pyramid of Histograms of Oriented Gradients), deep features, and an improved entropy-based feature. The improved entropy-based feature extraction method retains higher spatial distribution, resulting in correct region discrimination in segmented images.
- Lastly, a hybrid DL model (ILN-TL-DM) is presented for lung cancer classification, which is a combination of an improved LeNet model with Transfer Learning and a DeepMaxout model. These models are combined through soft voting, which improves the precision of lung cancer classification. Figure 1 represents the entire process of lung cancer classification.

### Preprocessing of CT image via adaptive Gaussian filtering

During preprocessing, the CT image $Img$ is filtered with an Adaptive Gaussian filtering^27^ method to eliminate noise and improve image quality. To improve the clarity of CT images, Gaussian filtering is utilized to eliminate high-frequency noise and achieve a smoother appearance. The filter works on a Gaussian function that gives different weights to neighboring pixels according to their distance from the central pixel. The two-dimensional discrete Gaussian filter can be expressed in Eq. (1).

1 $$\overline{G}\left( {\dot{x},\dot{y}} \right) = \frac{1}{{\sqrt {2\pi \tilde{\sigma }} }}{\text{e}}^{{\left( { - \frac{{\dot{x}^{2} + \dot{y}^{2} }}{{2\tilde{\sigma }^{2} }}} \right)}}$$

wherein, $\overline{G}\left( {\dot{x},\dot{y}} \right)$ signifies the value of the Gaussian filter at pixel position $\left( {\dot{x},\dot{y}} \right)$ and $\tilde{\sigma }$ signifies the standard deviation of the Gaussian distribution, which determines the amount of smoothing applied to the image. A larger $\tilde{\sigma }$ produces more smoothing. This filtering process effectively smooths out noise without deteriorating the significant image features, which are vital for subsequent processing. The outcome processed image, referred to as $Img^{Pre}$, is ready for further stages like segmentation and feature extraction with less noise and improved quality for correct analysis.

### Segmentation of preprocessed images via proposed attention-based ResU-Net architecture (P-ResU-Net)

The segmentation step of the suggested model is highly important for effectively separating the lung and tumor areas from the preprocessed CT images, $Img^{Pre}$. The traditional methods, like U-Net, treat all spatial regions equally and lack mechanisms to focus on clinically relevant areas, which often results in inaccurate or blurred segmentation boundaries. Moreover, the absence of residual connections in U-Net limits its depth and learning capacity, making it prone to vanishing gradients and performance degradation in deeper architectures. To address these limitations, this research employs the Proposed Attention-based ResU-Net (P-ResU-Net) model, which integrates the Proposed Residual Dual Channel Attention Block (RDCAB) and uses the proposed activation functions, including PReLU and d-SiLU functions, to improve feature representation across different intensity ranges. These enhancements collectively enable P-ResU-Net to achieve more precise and focused segmentation, better preserve boundary details for the effective segmentation of tumor regions within images, compared to traditional methods.

#### Traditional ResU-Net model

ResU-Net^28^ is a deep residual U-Net architecture that is used for semantic segmentation tasks. Residual learning is incorporated into the traditional U-Net model, which is popularly utilized in medical image segmentation. The standard ResU-Net architecture consists of an encoder-decoder model, where the encoder extracts the features of the image and the decoder maps the extracted features to produce the segmentation map. ResU-Net uses residual blocks rather than standard convolutional layers to enhance feature learning and gradient flow. Residual blocks are comprised of several convolutional layers with ReLU activation followed by batch normalization (BN). Two 3 × 3 convolutions are utilized in every layer in the conventional U-Net, but two pre-activated residual blocks replace them in ResU-Net to enhance feature dependencies.

However, despite its benefits, the traditional ResU-Net has some limitations, especially in terms of generalization capability when faced with image quality variations. As the depth of the network is increased and attention mechanisms are used, the problem of feature disappearance emerges, where the most important features might be lost behind multiple weightings during the process of attention mechanisms. This implies that as features are being balanced to highlight specific areas, some key details are lost in the process.

#### Proposed ResU-Net model

To overcome these constraints, the P-ResU-Net uses a Proposed Residual Dual Channel Attention Block (P-RDCAB). The block is placed after every residual block, enhancing the model’s capacity to emphasize and preserve key features and reject unnecessary ones. The P-RDCAB operates by paying attention to both channel attention and spatial attention, allowing the network to dynamically adapt to the significant features in the input image. With the help of the attention mechanism, the model can selectively enhance the most relevant features necessary for proper lung and tumor segmentation, downgrading the effect of less significant information.

**Fig. 2:**
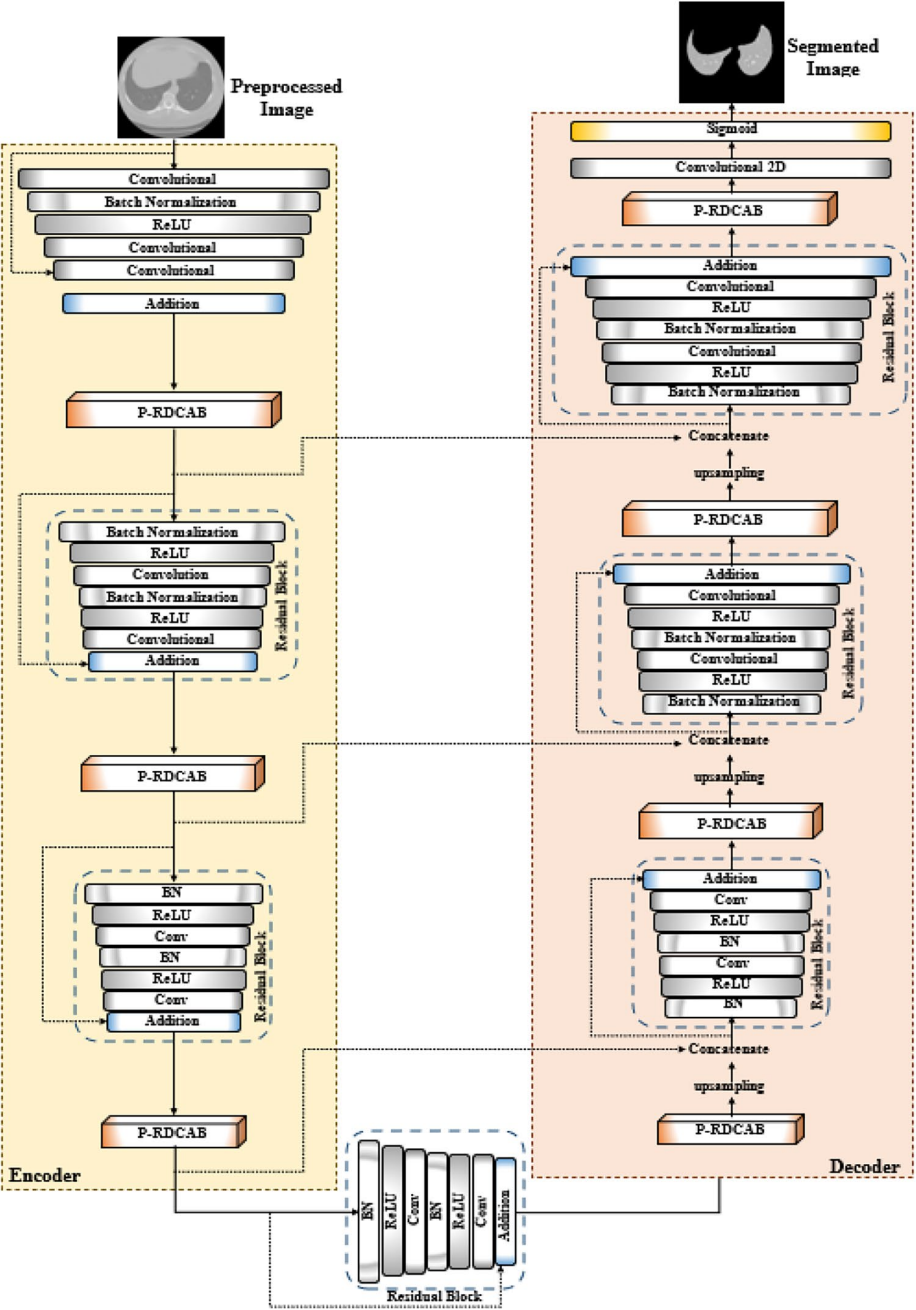
Architecture of P-ResU-Net.

Figure 2 depicts the structure of the P-ResU-Net model, which starts by passing the preprocessed image to the encoder block, which has convolutional layers that follow BN and ReLU activation. These are then fed to further convolutional layers. The result from the final convolution layer is added to the output of the previous layer, and this sum is fed into the P-RDCAB. The P-RDCAB improves the feature maps by highlighting important channels and suppressing less relevant ones. Thereafter, that output is inputted into the residual block, followed by another P-RDCAB. This is performed iteratively with each residual block preceded by a P-RDCAB, ensuring that the model keeps enhancing its feature maps.

Following processing through the encoding network, the segmentation map is generated by the decoder network. The decoder begins with a P-RDCAB, followed by upsampling layers. The output of the upsampling and the final P-RDCAB block from the encoder are combined and fed into a residual block. This is done several times in the decoder, with each refinement sharpening features until the final segmentation map is produced. The final segmentation output, $Img^{Seg}$ is generated after passing through a convolutional layer with a sigmoid activation function.

### Proposed residual dual channel attention block (P-RDCAB)

**Fig. 3:**
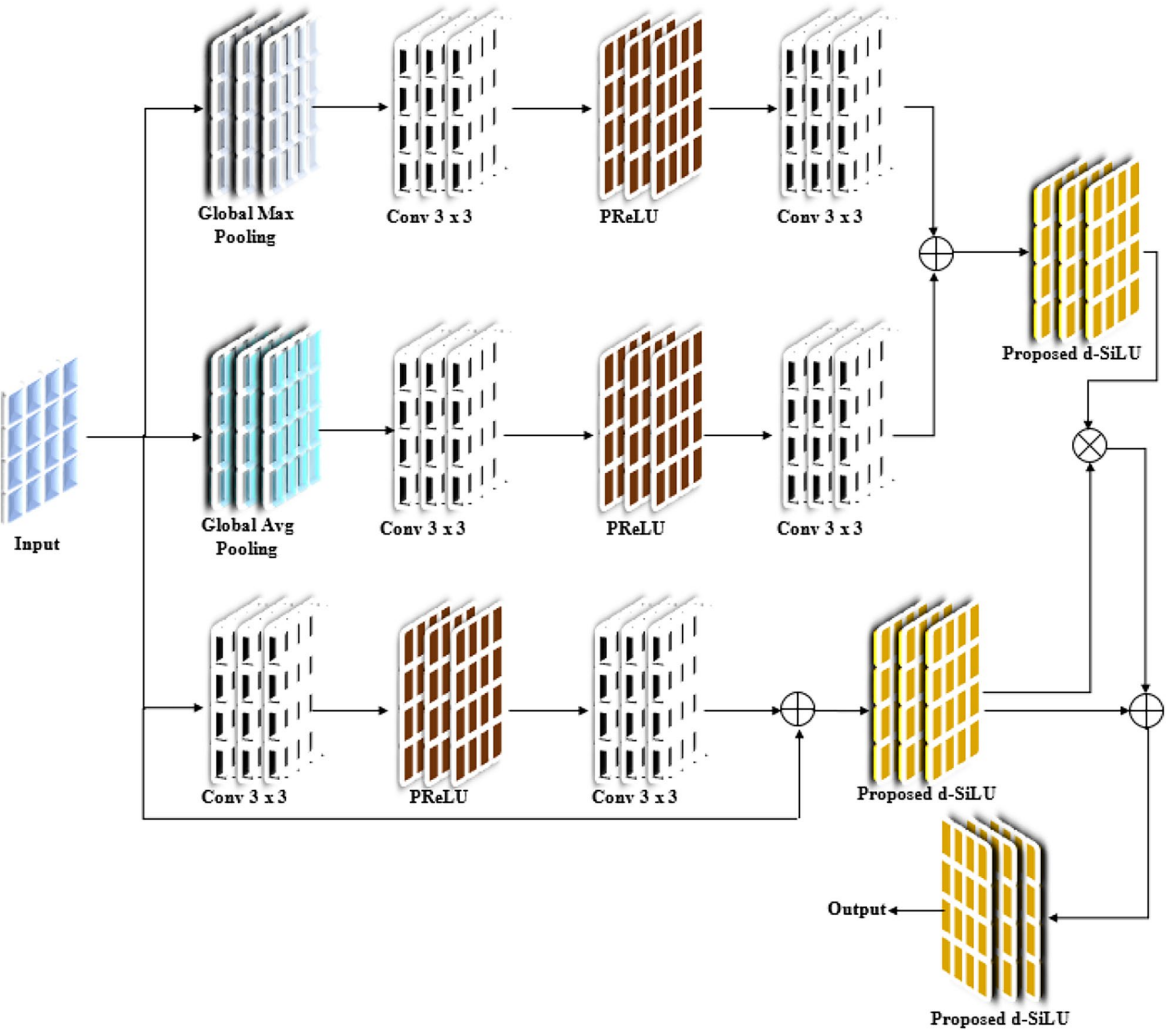
Architecture of P-RDCAB.

In the P-ResU-Net model, the attention blocks (through P-RDCAB) are incorporated into the residual blocks. Through this incorporation, the network can adaptively focus on the most significant areas of the CT image, thereby assisting the model in segmenting lung and tumor areas more precisely. The structure of P-RDCAB is depicted in Fig. 3. The P-RDCAB is made up of three parallel layers: Global Max Pooling (GMP), Global Average Pooling (GAP), and a 3 × 3 convolution. The outputs of GMP and GAP are fed through convolution layers and PReLU activations. These features are then combined, and their result is passed through a proposed d-SiLU activation function to further refine the features. The third parallel convolution layer is also passed through PReLU and convolution layers. The outputs of all three parallel branches are finally combined, and the output is passed through additional d-SiLU activations to get the outcome from the P-RDCAB.

### PReLU activation function

The PReLU activation function^29^ incorporates a learnable parameter of the negative slope and enables the model to make adjustments adaptively as per diverse feature distributions. For the task of segmentation, it is critical to accommodate different images based on dissimilar contrast and intensity. The PReLU function is defined in Eq. (2), where $$\overset{\lower0.5em\hbox{$\smash{\scriptscriptstyle\frown}$}}{x}_{i}$$ designates the input feature, and $$\alpha_{i}$$ signifies a trainable parameter that determines the slope for negative values. It helps in handling these variations more effectively, ensuring that the network remains flexible as well as robust during training and segmentation.

2 $$PReLU,f\left( {\overset{\lower0.5em\hbox{$\smash{\scriptscriptstyle\frown}$}}{x} } \right) = \left( {\begin{array}{*{20}c} {\overset{\lower0.5em\hbox{$\smash{\scriptscriptstyle\frown}$}}{x}_{i} ,} & {if\,\,\overset{\lower0.5em\hbox{$\smash{\scriptscriptstyle\frown}$}}{x}_{i} > 0} \\ {\alpha_{i} \overset{\lower0.5em\hbox{$\smash{\scriptscriptstyle\frown}$}}{x}_{i} ,} & {if\,\,\overset{\lower0.5em\hbox{$\smash{\scriptscriptstyle\frown}$}}{x}_{i} \le 0} \\ \end{array} } \right)$$

### Proposed d-SiLU activation function

In addition to PReLU, the proposed model incorporates the proposed d-SiLU (Derivative of Sigmoid Weighted Linear Units) activation function. The traditional d-SiLU activation function is usually employed to help optimize neural network weight parameters during gradient-descent updates. The standard form of d-SiLU is defined in Eq. (3), where $\hat{x}_{k}$ indicates input and $\alpha_{k} \left( S \right)$ indicates output. However, this function has the demerit of having the vanishing gradient problem in deep networks, thus being inefficient for large models with numerous layers. It is also not suitable to handle changing data, hence limiting its performance in image segmentation.

3 $$\alpha_{k} \left( S \right) = \alpha \left( {\hat{x}_{k} } \right) \cdot \left( {1 + \hat{x}_{k} \left( {1 - \alpha \left( {\hat{x}_{k} } \right)} \right)} \right)$$

An improved d-SiLU activation function is proposed to address the above limitations, which is calculated in Eq. (4). Where $SF\left( {\hat{x}} \right)$ indicates the sigmoid function, defined in Eq. (5). $HTF_{{\hat{x}}}$ signifies the hyperbolic tangent function using Eq. (6). The suggested function incorporates a dynamic scaling factor that improves gradient flow and responds better to the input features, enhancing learning efficiency.

4 $$F\left( {\hat{x}} \right) = SF\left( {\hat{x}} \right) * \left( {1 + \hat{x}\left( {1 - SF\left( {\hat{x}} \right)} \right)} \right)$$

5 $$SF\left( {\hat{x}} \right) = \frac{1}{{1 + \exp \left( { - HTF_{{\hat{x}}} } \right)}}$$

6 $$HTF_{{\hat{x}}} = \frac{{e^{{\hat{x}}} - e^{{ - \hat{x}}} }}{{e^{{\hat{x}}} + e^{{ - \hat{x}}} }}$$

Thus, the P-ResU-Net has several important benefits: it employs P-RDCAB with channel and spatial attention to highlight significant features and suppress non-significant ones. Residual learning maintains features across the network, avoiding loss in deeper layers. The application of PReLU and d-SiLU activation functions also increases flexibility, enabling the model to adjust to different image conditions, which improves segmentation accuracy.

### Extracting sophisticated features from segmented images

Following segmentation, extracting several significant features from the segmented image, $Img^{Seg}$ to improve the accuracy of classification. They are Local Gabor Transitional Pattern (LGTrP), Pyramid of Histograms of Oriented Gradients (PHOG), deep features of networks such as AlexNet and ResNet50 and improved entropy-based features. All of these features are essential for enhancing the performance of the segmentation model by giving detailed information to be processed further in the classification stage.

#### Local gabor transitional pattern (LGTrP), $f_{Img}^{LGTrP}$

In this proposed lung cancer classification framework, Local Gabor Transitional Pattern (LGTrP), $f_{Img}^{LGTrP}$^30^ is used to extract robust texture features from the segmented lung region, $Img^{Seg}$. This descriptor combines the spatial-frequency sensitivity of Gabor filters with the structural encoding power of Local Transitional Pattern (LTP) to efficiently detect local texture variations corresponding to abnormal tissue patterns that are predictive of lung cancer.

The process is initiated with the use of Gabor filters, which are well known to extract multi-orientation and multi-resolution features and hence are more appropriate for the analysis of texture in medical images. The mathematical definition of a 2D Gabor filter is expressed in Eq. (7), where $$\overset{\lower0.5em\hbox{$\smash{\scriptscriptstyle\frown}$}}{s} \left( {x,y} \right)$$ signifies a complex sinusoid and $$\overset{\lower0.5em\hbox{$\smash{\scriptscriptstyle\frown}$}}{w} \left( {x,y} \right)$$ signifies a Gaussian-shaped envelope. These filters highlight critical texture patterns in lung regions that may correspond to cancerous tissue.

7 $$G\left( {x,y} \right) = \overset{\lower0.5em\hbox{$\smash{\scriptscriptstyle\frown}$}}{s} \left( {x,y} \right)\overset{\lower0.5em\hbox{$\smash{\scriptscriptstyle\frown}$}}{w} \left( {x,y} \right)$$

Following Gabor filtering, LTP is used to further improve fine-grained structural encoding. In contrast to Local Binary Pattern (LBP), LTP examines the center pixel and two neighbors in a particular direction to analyze the transition of intensity, an important indicator of abnormal tissue structure in images. The LTP code is computed in Eq. (8), wherein, $G_{c}$ indicates the intensity of the center pixel, $G_{l1}$ and $G_{l2}$ are intensities at radii $r1$ and $r2$ from the center in direction $l$.

8 $$LTP_{l,r1,r2} \left( {x_{c} ,y_{c} } \right) = \sum\limits_{l = 0}^{l - 1} {\overset{\lower0.5em\hbox{$\smash{\scriptscriptstyle\frown}$}}{s} \left( {G_{l1} - G_{c} } \right) \otimes } \left( {G_{l2} - G_{c} } \right) * 2^{l}$$

To preserve spatial context, the segmented lung image is separated into several sub-regions and LTP histograms are calculated for each. The final LGTrP feature vector is created by concatenating these histograms. Through the combination of directional filtering and structural pattern encoding, LGTrP delivers a strong textural abnormality representation in lung tissue to improve the classification capability in identifying cancerous areas.

#### PHOG (pyramid of histogram of oriented gradients), $f_{Img}^{PHOG}$

The Pyramid of Histogram of Oriented Gradients (PHOG)^31^ is applied to extract spatial and gradient-based features of the segmentation image, $Img^{Seg}$ in the feature extraction phase. PHOG builds upon the standard HOG descriptor with the advantage of using multi-resolution representations to increase detailed spatial descriptions across scales. PHOG extracts the features based on dividing an image into segments in different regions across pyramid levels and applying the analysis of the regions based on gradient orientations. The process starts with the application of a Sobel operator to estimate the gradient magnitude $\dot{G}$ and orientation of gradient C for every pixel of the image, as defined by Eqs. (9) and (10), respectively, wherein, $\dot{G}_{p}$ and $\dot{G}_{q}$ represents gradients in the direction $x$ and direction $y$, respectively.

9 $$\dot{G} = \sqrt {\dot{G}_{p}^{2} + \dot{G}_{q}^{2} }$$

10 $$\dot{C} = \arctan \left( {\frac{{\dot{G}_{p} }}{{\dot{G}_{q} }}} \right)$$

After computing gradient information, the image is sectioned into cells in a cell grid. There are cells into which each histogram bin for every cell sums the gradient magnitudes. Every histogram bin represents some orientation. PHOG expands by representing the image at several pyramid levels, beginning with big areas at the bottom level and further splitting them up into smaller ones at subsequent levels. The gradients are calculated and added over the areas, and the histograms formed as a result are concatenated together to create a feature vector. Thus, the PHOG descriptor captures both local and global gradient patterns and their spatial arrangement at various scales, which is especially useful for separating tumor areas from regular lung tissue in CT scans.

#### Deep features (ResNet 50 and AlexNet), $f_{Img}^{Deep}$

Segmented CT images, $Img^{Seg}$ are processed to extract Deep features, $f_{Img}^{Deep}$ through the application of two popular pre-trained CNNs, namely AlexNet and ResNet50. These models are selected due to their excellent ability to extract high-level features from images. Both AlexNet and ResNet50 allow the model to learn applicable features without needing large amounts of task-specific data. Through the use of these robust networks, the system can focus towards retrieving intricate features that are strongly predictive of lung cancer.

##### AlexNet

AlexNet^32^ is a pioneering DL architecture that is known for its achievement in Image classification tasks. It consists of several convolutional as well as fully connected layers. The convolutional layers extract key spatial features like edges, textures, and intricate patterns from the images, $Img^{Seg}$. After convolution, Max pooling layers are applied to decrease the spatial dimensions of the feature maps, retaining the most critical information. AlexNet utilizes ReLU activation functions to achieve non-linearity, as well as dropout layers for preventing overfitting when training. Fully connected layers at the end of the network sum up the features extracted and decide based on learned representations. By utilizing AlexNet, it extracts deep features that represent the complex structures, hence proving efficient for lung cancer classification.

##### ResNet 50

In this paper, ResNet50^33^ is utilized for deep feature extraction of segmented CT images based on its residual learning architecture. ResNet50 is architected to overcome the vanishing gradient issues related to conventional deep networks by utilizing residual connections. These connections enable the network to learn the “difference” or “residual” between the input and the output instead of learning the complete transformation in one step. This allows the network to concentrate on learning the more intricate details in the data while retaining the information from previous layers. The network has 50 convolutional layers arranged in residual blocks, with each block refining and sharpening features developed from the input image. There follows a fully connected (FC) layer used to accumulate and weigh the learned features by the network. This FC layer allows the model to classify the image on the basis of the hierarchical, complex features obtained from the segmented lung CT images, $Img^{Seg}$. Utilizing ResNet50, the system also gets the advantage of its deep architecture and residual learning that enhances feature extraction. The dimension of the extracted deep features is overly large; hence, the Principal Component Analysis (PCA) approach is applied to the deep features. This PCA is a dimensionality reduction technique that transforms a set of possibly correlated variables into a set of linearly correlated variables. This allows for a significant reduction in the dimensionality while retaining most of the important information, crucial for precise and reliable classification.

#### Improved entropy-based features, $f_{Img}^{En}$

During the feature extraction step, improved entropy (IEn) based features, $f_{Img}^{En}$ are derived from the segmented image, $Img^{Seg}$ to better represent spatial distribution information. Entropy in image processing quantifies the level of randomness in the image, representing the complexity of pixel intensity distributions. Conventional entropy, namely Shannon entropy, is first used on the image, but this method is limited because it only looks at the frequency distribution of pixel intensities without accounting for the spatial distribution of these intensities. In conventional entropy, Shannon entropy^34^ is defined as per Eq. (11), where $p\left( m \right)$ signifies the probability of occurrence of the intensity value $m$ and $M$ signifies the number of separate gray levels in the image $\left( {M = 2^{t} } \right)$, with $t = 8$ being the number of bits per pixel (for grayscale images). $En\left( {Img^{Seg} } \right)$ represents the image entropy, which is a measure of how much information is present in the image. The value of this entropy between 0 and $t$ gives the amount of information present in the image.

11 $$En\left( {Img^{Seg} } \right) = - \sum\limits_{m = 0}^{M - 1} {p\left( m \right)\log_{2} \left( {p\left( m \right)} \right)}$$

However, the traditional entropy equation addresses only the frequency of intensity levels throughout the image and does not take into consideration the spatial arrangement of those intensity levels. (i.e., it does not address the structure of pixels within the image, which is most important in addressing the structure and context of regions of interest). Therefore, there is a need for a more complex method of entropy that includes spatial information.

To overcome the deficiencies of traditional entropy, this paper presents an improved entropy model. The new entropy is a weighted sum of Shannon entropy and Normalized Entropy (NE)^35^, which is a more effective description of the spatial characteristics of the image. The new improved entropy formula is expressed in Eq. (12), wherein $W_{En}$ signifies a weight factor, $En\left( {Img^{Seg} } \right)$ indicates the Shannon entropy, which is calculated in Eq. (11) and $NEn\left( {Img^{Seg} } \right)$ signifies normalized entropy of the image.

12 $$IEn\left( {Img^{Seg} } \right) = W_{En} \cdot En\left( {Img^{Seg} } \right) + \left( {1 - W_{En} } \right) \cdot NEn\left( {Img^{Seg} } \right)$$

The weight $W_{En}$ is computed by a reverse sigmoid function of entropy so that the system can dynamically adjust the impact of Shannon entropy depending on the local characteristics of the image using Eq. (13). This operation controls the contribution of Shannon entropy to the overall process of feature extraction so that it becomes more sensitive to local image variations.

13 $$W_{En} = 2 \times \left( {1 - \frac{1}{{1 + \exp \left[ {En\left( {Img^{Seg} } \right)} \right]}}} \right)$$

In Eq. (12), the Normalized Entropy, $NEn\left( {Img^{Seg} } \right)$ is computed to obtain a more detailed measurement of entropy considering the spatial intensity distribution of the pixels. Normalized entropy formula is given by Eq. (14). where $En_{1k} \left( {Img^{Seg} } \right)$ signifies the first-order entropy of the image, which is calculated in Eq. (15), $N_{m}$ signifies the number of distinct pixel values, $P_{I}^{1} \left( L \right)$ define the probability of $L$ th value among all $N_{m}$ as per Eq. (16). where, $M_{L}$ and $M_{J}$ indicates the frequency value of $L$, $J$ pixels. This computation helps to capture finer details within the image, which are essential for distinguishing between different regions in the Segmented image.

14 $$NEn\left( {Img^{Seg} } \right) = \frac{{En_{1k} \left( {Img^{Seg} } \right)}}{{\log_{2} \left( {N_{m} } \right)}}$$

15 $$En_{1k} \left( {Img^{Seg} } \right) = - \sum\limits_{L = 1}^{{N_{m} }} {P_{I}^{1} \left( L \right)} \cdot \log_{2} \left( {P_{I}^{1} \left( L \right)} \right)$$

16 $$P_{I}^{1} \left( L \right) = \frac{{M_{L}^{\left( I \right)} }}{{\sum\limits_{J - 1}^{{N_{m} }} {M_{J}^{\left( I \right)} } }}$$

The most important benefit of the IEn method is that it can dynamically modify the impact of Shannon entropy according to the local features of the image. The weight factor $W_{En}$ ensures that the calculation of entropy is not fixed but is rather modulated based on local features around each pixel. This allows the suggested entropy to be better in terms of modeling spatial variation within the image, which is an important consideration in tasks such as lung cancer classification. The dimension of the extracted features is shown in Table 2.

**Table 2:** Dimension of features.

Total dimension of the extracted feature = 201	
Features	Dimension
LGTrP	1 × 50
PHOG	1 × 50
Improved entropy	1 × 1
Deep features	1 × 100

The LGTrP is effective in capturing fine-grained local texture variations through Gabor-filtered transitional patterns, aiding in the identification of subtle textural irregularities often associated with tumor regions. The PHOG provides a multi-scale representation of edge orientation and spatial layout, which is crucial for preserving structural and shape-related information for accurate classification. Deep features extracted from pre-trained models such as AlexNet and ResNet50 enable the learning of high-level semantic representations without requiring large amounts of domain-specific data, improving generalization. Additionally, the improved entropy-based features quantify irregularity and randomness in pixel intensity distributions, enhancing sensitivity to subtle changes in image texture and structure. By integrating LGTrP, PHOG, deep features, and entropy-based features, the feature extraction process becomes more comprehensive and robust, leveraging the complementary strengths of handcrafted and deep learning-based features. This fusion enables the model to better distinguish between cancerous and non-cancerous tissues. Finally, all extracted features are concatenated and merged to create a combined feature set, $f_{Img} = \left[ {f_{Img}^{LGTrP} \,\, \cup f_{Img}^{PHOG} \, \cup \,f_{Img}^{Deep} \, \cup \,f_{Img}^{En} } \right]$ which is then input into the classification model. This enables the system to leverage both texture-based features and higher-level features extracted by deep learning models, allowing for more precise lung cancer classification.

### Classification via improved LeNet with TL and deep maxout

**Fig. 4:**
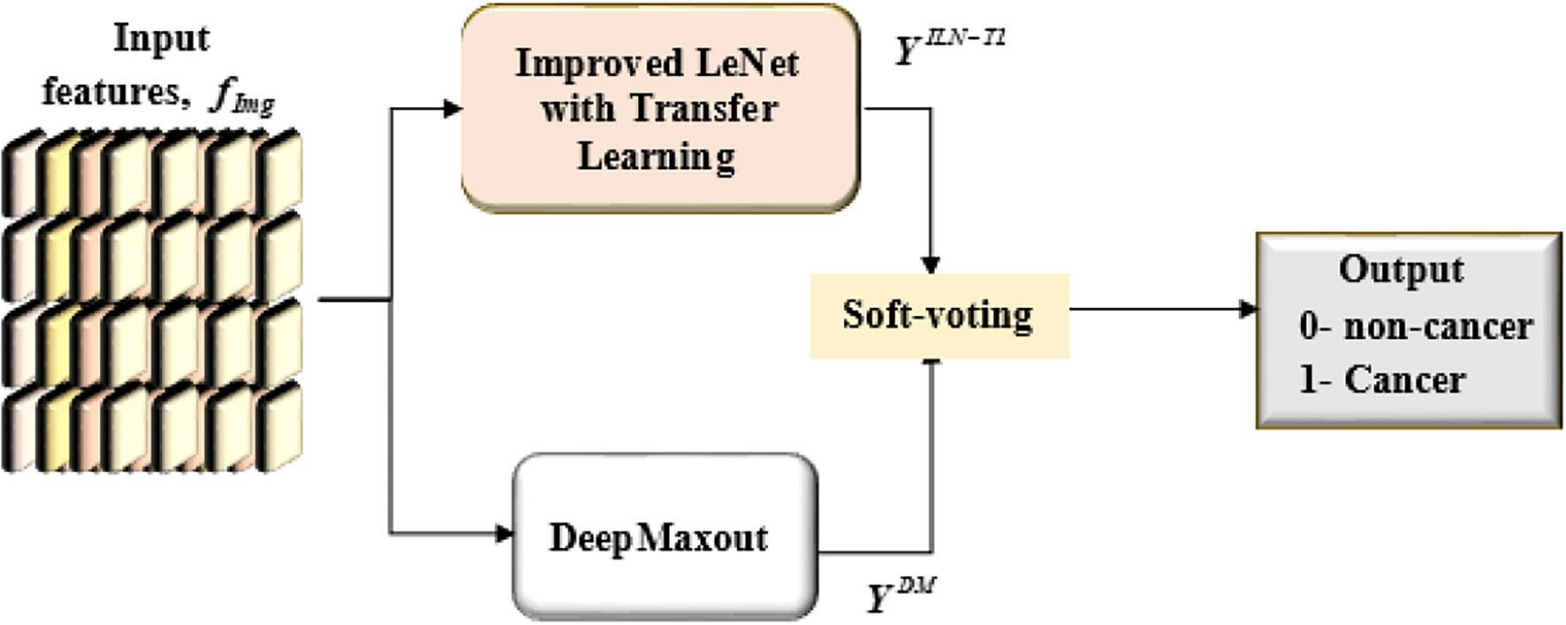
Lung cancer classification process.

The classification stage employs a Hybrid DL model, which integrates two sophisticated architectures, namely Improved LeNet with Transfer Learning (ILN-TL) and DeepMaxout (DM). Specifically, the Improved LeNet model leverages the strength of transfer learning and the architectural enhancements contribute to improving the generalization and reducing the overfitting. On the other hand, the DeepMaxout excels in learning complex, high-capacity nonlinear representations through its Maxout activation function. This makes DM particularly effective in distinguishing subtle differences in tumor features. Leveraging the complementary strengths of both ILN-TL and DM models significantly contributes to enhancing the discriminative ability. This integrated model aims to leverage the advantages of both architectures and improve the accuracy of the classification of lung cancer based on the extracted features, $f_{Img}$. The final classification result is obtained using a soft voting approach, in which both models’ predictions are combined, resulting in a binary output of 0 for non-cancer and 1 for cancer. Figure 4 displays the overall process of the classification phase.

#### Improved LeNet with TL (ILN-TL)

The ILN-TL is intended to improve lung cancer classification by harnessing the strength of deep learning and pretrained models. This is a method of leveraging a pretrained model, VGG-16^36^, to extract features from the input image. The features learned by the pretrained model are fed improved LeNet model to create the final classification result. The ILN-TL’s output is represented as $Y^{ILN - TL}$.

### Conventional LeNet model

**Fig. 5:**
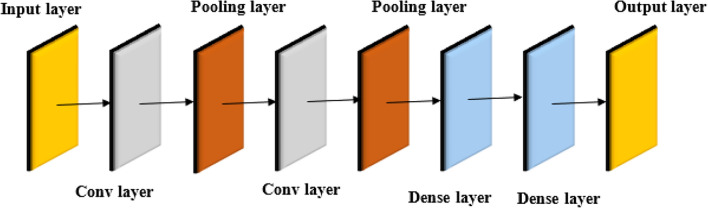
Architecture of the conventional LeNet model.

The LeNet model^37^ was initially designed for image classification, which consists of a relatively simple architecture with only 7 layers. It includes an input layer, which receives the image, followed by convolutional layers. Then, pooling layers are employed to downsample the feature maps, reducing their spatial dimensions while preserving important information. The outcome of the pooling layers is then fed into fully connected layers, which merge the extracted features to arrive at the final classification decision. The model concludes with a classification layer that produces the predicted class. The architecture of the conventional LeNet model is shown in Fig. 5.

Although the traditional LeNet, with its shallow 7-layer architecture, which is struggles with lung cancer classification. Its shallow depth is not deep enough to learn hierarchical and abstract representations, which are necessary for detecting sophisticated patterns of medical images. This inadequacy in complexity reduces its performance in extracting subtle image variations, leading to lower performance in cancer detection. Additionally, the conventional LeNet model does not leverage pre-trained knowledge from datasets, which limits its generalization capability. To address these limitations, this research introduces an Improved LeNet model that incorporates the concept of transfer learning. The architectural enhancements include the addition of a Gaussian normalization layer and the integration of VGG-16 as a feature extractor, enabling the model to benefit from pre-trained representations. These modifications significantly improve the model’s ability to distinguish subtle texture variations, thereby enhancing classification accuracy.

The ILN-TL model enhances the original LeNet model by adding VGG-16, a pre-trained model, as a feature extractor. TL allows the model to benefit from the knowledge that VGG-16 has already accumulated on large datasets such as ImageNet. Instead of beginning from scratch, the model leverages VGG-16’s capability to learn high-level, hierarchical features from a huge, heterogeneous dataset and then modifies these features to suit lung cancer classification. This improvement gives the network the ability to learn abstract features and complex patterns that are important in the detection of cancer cells in medical images. In addition, the improved LeNet model contains extra convolutional layers, Depthwise Separable convolutions, and pooling to further enhance the model’s ability to detect meaningful features, enhancing its performance and accuracy in this particular task.

### Transfer learning

TL enables the Improved LeNet model to use the VGG-16 trained on ImageNet for classifying lung cancer. The model uses features like edges and textures from ImageNet to learn, without beginning from scratch, saving time and computational power. The initial layers of VGG-16 are able to detect universal features, which can be fine-tuned using lung cancer images to adapt to the specific task and enhance performance without needing a large amount of labeled data.

### VGG 16 model

Existing advanced architectures, such as EfficientNet and Vision Transformers, involve more complex training procedures and numerous hyperparameters, making model tuning time-consuming and computationally intensive. Moreover, these models often function as black boxes, offering limited interpretability, an essential requirement in medical image analysis. To address these challenges, this research employs the VGG-16 model as a pre-trained feature extractor. Specifically, the VGG-16 is known for its stability, robustness, and lower sensitivity to hyperparameters, making it more efficient in terms of training resources while still providing strong and interpretable feature representations. The VGG-16 model is the CNN, with a total of 16 layers that comprise 1 input layer, 5 convolutional layers, 3 fully connected layers, 3 max pooling layers, ReLU activation layers, 2 normalization layers, 2 dropout layers and a final classification layer. The architecture is simple in terms of design, yet it is highly capable of learning large datasets. The core of VGG-16 lies in its use of 3 × 3 convolution filters in all the convolutional layers. Every convolutional layer learns progressively more complex features. The model applies max pooling after every pair of convolutions to decrease spatial dimensions and boost computational efficiency. The fully connected layers (fc6 and fc7) are where deep features are extracted so that the model can learn complex patterns required for classification tasks. The Softmax output layer gives the classification result, showing the probability of each class. VGG-16 has become a standard for feature extraction because it is simple, uniform, and can learn rich, hierarchical features from large datasets.

### Structure of ILN-TL

**Fig. 6:**
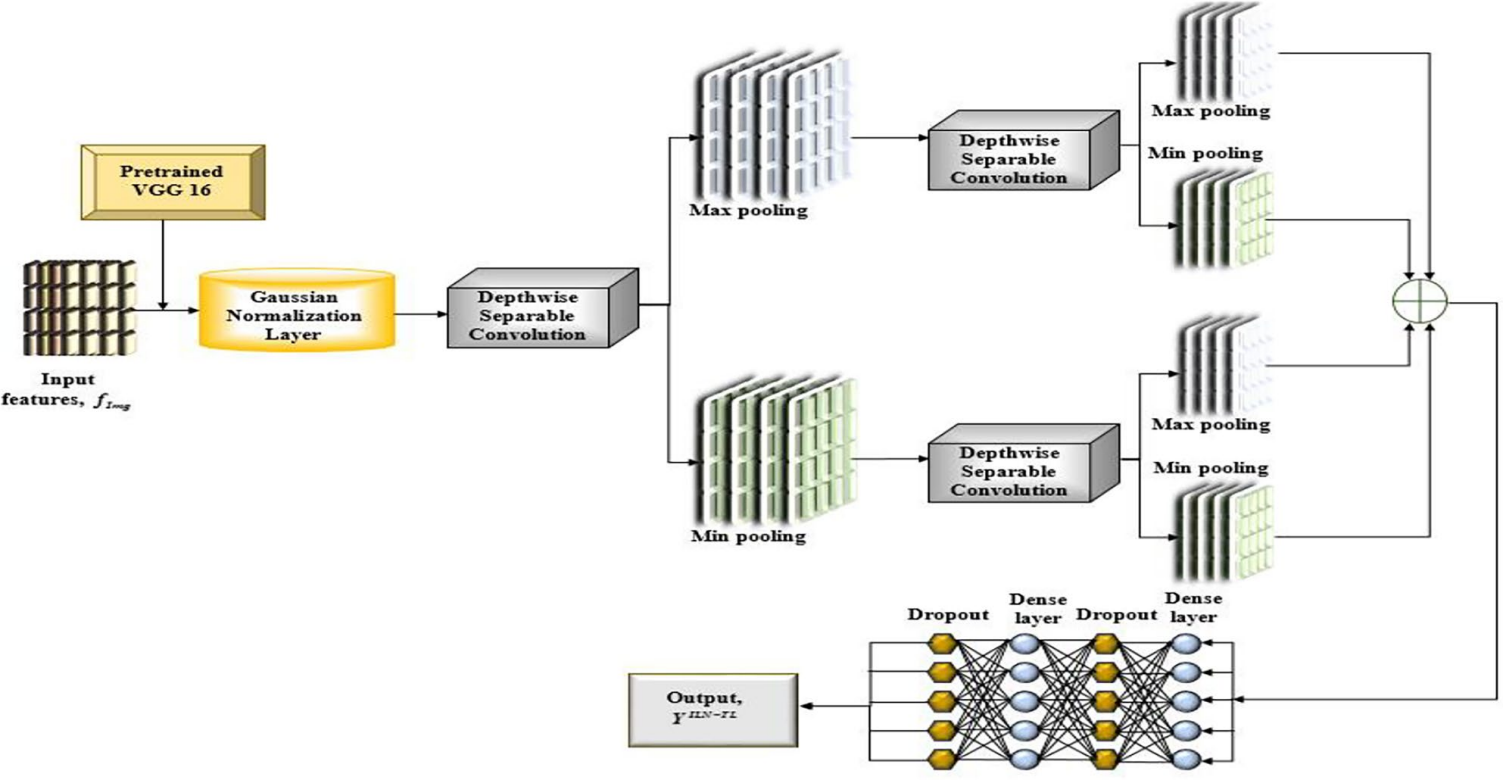
Structure of the improved LeNet model.

**Fig. 7:**
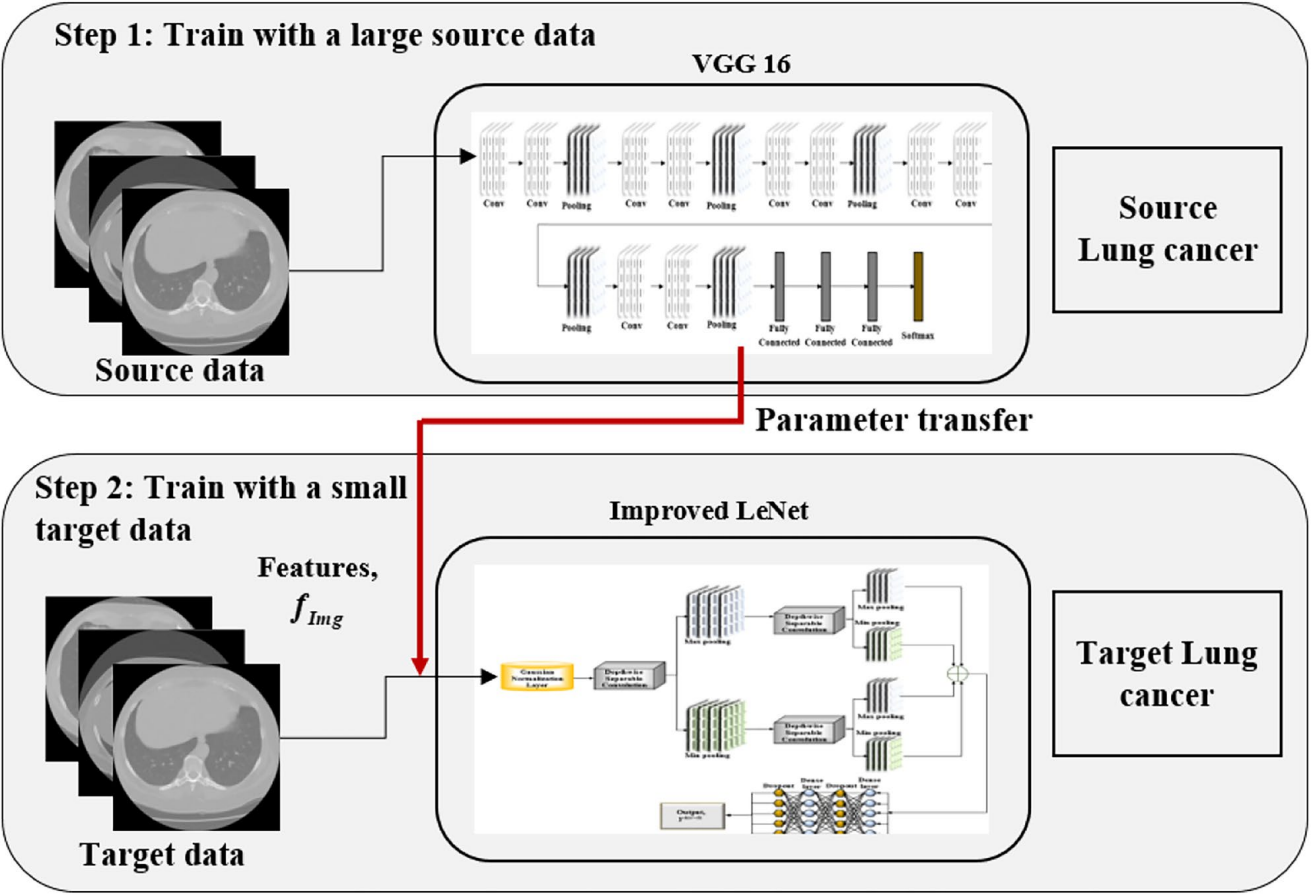
Structure of ILN-TL model.

The ILN-TL model enhances the basic LeNet architecture by using the VGG-16 pre-trained model as the first feature extractor. The improved LeNet model is shown in Fig. 6. The features extracted from VGG-16 are initially sent through a Gaussian normalization layer, which normalizes the features prior to further processing. The normalization helps stabilize the features into a consistent range to enhance the efficiency of the training process. Upon normalization, the features undergo Depthwise Separable Convolutional layers, designed to decrease the parameters and computational complexity compared to normal convolutions. The outcome of the Depthwise Separable Convolutional layers is subsequently passed through a sequence of max and min pooling layers. This two-stage pooling procedure captures both major and minor features in the input image, and this improves the model’s capability to identify diverse patterns. The features are subsequently passed through some dense layers, which fuse the information that has been extracted and make the final decision in terms of the classification. Dropout layers are used after every dense layer to avoid overfitting. The last output layer gives a binary classification, which is denoted as $Y^{ILN - TL}$. Figure 7 depicts the structure of the ILN-TL model.

### Depthwise separable convolutional layer

In this model, Depthwise Separable Convolutions are employed. This method significantly decreases the number of parameters and computational expense by using a convolution operation independently on each input channel, rather than conducting a full convolution on all channels. This means fewer computations and parameters, making the model more efficient and faster to train, particularly when dealing with large datasets like medical images.

### Gaussian normalization layer

The Gaussian normalization^38^ layer normalizes the VGG-16 model-extracted features. Normalization is defined as per Eq. (17), where, $\sigma$ signifies the standard deviation of the feature values. $X$ signifies the input feature, and $N_{Z}$ is the normalized feature, $\mu_{L}$ indicates the Lehmer mean of the feature values using Eq. (18), here, the value of $Q$ is taken as 2.

17 $$N_{Z} = \frac{{\left[ {\frac{{X - \mu_{L} }}{3 \times \sigma } + 1} \right]}}{2}$$

18 $$\mu_{L} = \frac{{\sum\limits_{i = 1}^{N} {x_{i}^{Q} } }}{{\sum\limits_{i = 1}^{N} {x_{i}^{Q - 1} } }}$$

This normalization assists with stabilizing training and enhancing convergence by making certain that the values of the feature are uniformly distributed.

Therefore, the proposed model employs a pre-trained VGG-16, allowing it to acquire rich, hierarchical features from less data. Adding convolutional and Depthwise layers increases model depth, allowing it to capture complex patterns. Depthwise Separable Convolutions decrease parameters for quicker processing, while dropout and Gaussian normalization enhance training robustness and stability.

#### Deep maxout

During the classification stage, the Deep Maxout^39^ model works towards significantly enhancing the accuracy in classifying the lung cancer further. It has used Deep Maxout Networks (DMN), a form of activation function that focuses on maximizing the deep neural networks’ learning procedure. The key characteristic of the Maxout activation function is its ability to allocate a non-zero slope both to positive terms and negative terms, which provides it with an added degree of flexibility and responsiveness for intricate classification tasks. The Maxout function helps the network avoid vanishing gradients and enables the model to acquire deeper representations of the data by changing the slopes. This is very useful in keeping hidden layer units from entering into unwanted modes that may cause poor performance. The outcome of the Deepmaxout model is represented as $Y^{DM}$. Table 3 shows the hyperparameter settings of the model.

**Table 3:** Hyperparameters of the classifier.

Model	Hyperparameters
Improved Lenet	Input layer:1 GaussianNormalizationLayer:1, DepthwiseConv2D layer1: kernel (5,5), activation = ‘relu’, Fully connected layer1: hidden neuron = 128, Activation function = relu optimizer = ‘adam’, loss = 'categorical cross-entropy’, metrics = [‘accuracy’], batch size = 64, validation split = 0.2, learning rate = 0.001, Epoch = 50
Deep maxout	Batch size = 32 Learning rate = 0.01 Convolution layer1: Batch Normalization1 Pooling layer1: k size = 2 Activation function = softmax Epoch = 50

#### Soft voting

In this paper, soft voting^40^ is used in the classification stage to merge the outputs of the ILN-TL model and the DM model. In this soft voting, class labels are forecasted by the forecasted probabilities $p$ for every classifier. The approach is useful if classifiers are well-calibrated. Soft voting allows both models to contribute proportionally to the final prediction based on their predicted probabilities. This approach helps mitigate the risk of one model dominating the outcome due to overconfidence in uncertain samples, resulting in a more balanced and reliable classification. Prediction is computed in Eq. (19), where $\hat{w}_{j}$ signifies the weight assigned to the $j$-th classifier, here, the weight is assigned as 0.5 and $p_{ij}$ indicates the predicted probability of class $i$ from the $j$-th classifier, $c$ is the number of classifiers, $\left( {c = 2} \right)$.

19 $$Y = \arg \mathop {\max }\limits_{i} \sum\limits_{j = 1}^{c} {\hat{w}_{j} p_{ij} }$$

The final result is achieved by integrating the result from the improved LeNet with transfer learning, $Y^{ILN - TL}$ and the DeepMaxout model, $Y^{DM}$ using soft voting, where “0” designates “non-cancer” and “1” designates “cancer”.

## Results and discussion

### Simulation procedure

The Proposed Lung Cancer Classification using CT images was implemented using PYTHON 3.7. Further, the Processor exploited was “AMD Ryzen 5 3450U with Radeon Vega Mobile Gdx 2.10 GHz and the Installed RAM size was 16.0 GB”. Moreover, the analysis of Lung Cancer Classification was conducted using Luna16 dataset^41^.

### Dataset description

This dataset utilized the “Creative Commons Attribution 3.0 Unported License.” It encompasses a total of 888 CT scans. The Cancer class includes 589 scans, and the non-cancer class designates 299 scans. Also, this dataset employed 445 patients, where 306 patients for cancer and 139 patients did not have cancer. This database comprises comments which were composed through a two-phase comment procedure via 4 knowledgeable radio therapists. All radio therapists marked lesions they established as “non-nodule, nodule < 3 mm, and nodules >  = 3 mm.”

In this examination, we utilized a total of 2336 instances. The non-cancer class comprises 1112 instances, and Cancer contains 1224 instances. The training and testing details of the Luna16 dataset are shown in Table 4.

**Table 4:** Training and testing details of the dataset.

Training data (%)	Number of training images	Number of testing images
60	1401	935
70	1635	701
80	1868	468

### Performance analysis

An exhaustive comparative estimation was performed to analyze the efficiency of ILN-TL-DM in comparison to traditional strategies. This assessment encompasses a wide range of performance measures, including “Accuracy, Precision, Sensitivity, Specificity, F-Measure, MCC, NPV, FNR and FPR”. Moreover, the Ablation Study, ROC Curve Analysis and Statistical Evaluation were carried out to evaluate the effectiveness of the ILN-TL-DM model. Further, the ILN-TL-DM approach is compared against state-of-the-art methods like 3D AG-Net^26^, MFDNN^24^ and SCNN-PNN^42^ as well as the traditional approaches such as LeNet, DenseNet, Parallel CNN, LSTM and Deep Maxout. The ILN-TL-DM and traditional strategies were investigated using the Luna16 dataset.

### Preprocessing analysis

**Fig. 8:**
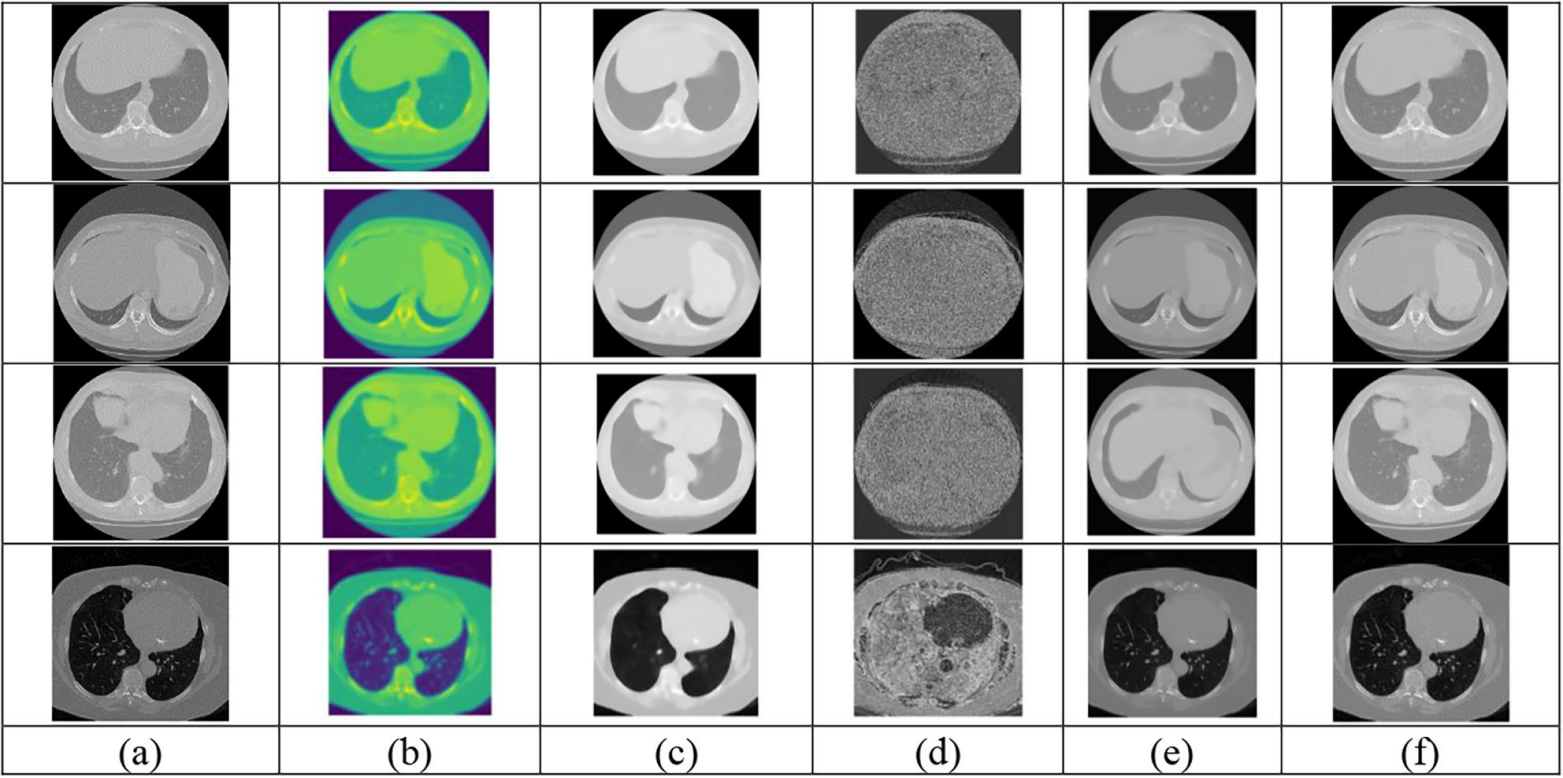
Pre-processed outcomes (**a**) Input Images (**b**) Gaussian filtering (**c**) Median filtering (**d**) Non-local means filtering (**e**) Wavelet denoised filtering and (**f**) Adaptive Gaussian Filtering.

Figure 8 displays the Sample Images alongside the results of Gaussian filtering, Median filtering, non-local means filtering, wavelet denoised filtering and adaptive Gaussian filtering based pre-processed images.

#### Analysis on PSNR and SSIM

Image quality has been assessed using metrics such as PSNR and SSIM. SSIM offers a more thorough assessment of image integrity than PSNR, which concentrates on pixel discrepancies. In this study, the effectiveness of the adaptive Gaussian filter is contrasted with that of well-known pre-processing techniques, including the Gaussian filter, median filter, Wavelet filter, and non-local means filter. Table 5 presents a comparative analysis of different image filtering methods based on PSNR and SSIM values. Among all methods, the Adaptive Gaussian filter achieves the highest performance with a PSNR of 37.378 dB and an SSIM of 0.935, indicating excellent noise reduction and structural preservation. On the other hand, the existing methods like the Gaussian filter follow closely with a PSNR of 35.270 dB and SSIM of 0.916, the Wavelet filter provides a PSNR of 31.279 dB, and SSIM of 0.903, the Median filter shows a lower PSNR of 27.822 dB and SSIM of 0.819, and the Non-local Means filter performs with the lowest PSNR of 17.599 dB and SSIM of 0.717, indicating significant information loss. Overall, the results emphasize that the Adaptive Gaussian filter is the most suitable for enhancing CT images prior to the classification of lung cancer.

**Table 5:** Analysis on PSNR and SSIM.

Methods	PSNR (db)	SSIM
Wavelet filter	31.279	0.903
Non-local means	17.599	0.717
Median filter	27.822	0.819
Gaussian filter	35.270	0.916
Adaptive Gaussian filter	37.378	0.935

### Segmentation analysis

**Fig. 9:**
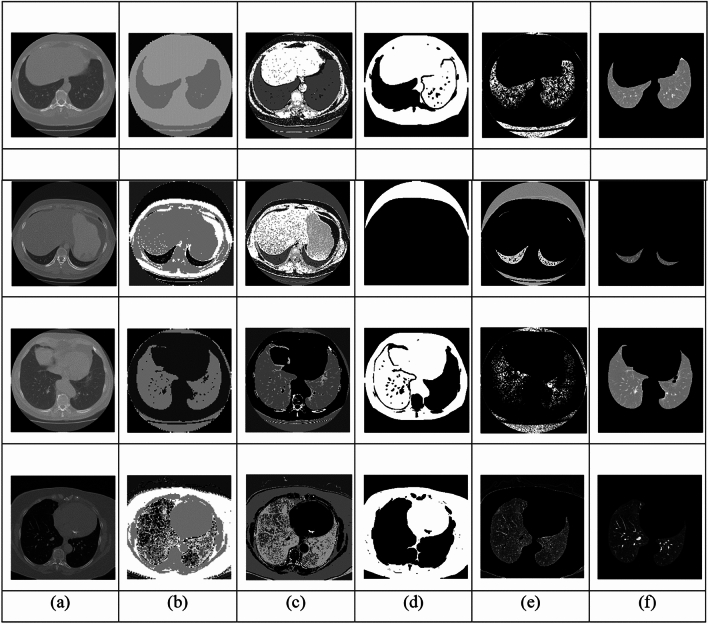
Segmented Results (**a**) Input Images (**b**) BIRCH (**c**) K-Means (**d**) Region Growing (**e**) Conventional Res U-Net and (**f**) P-ResU-Net.

Figure 9 illustrates the Sample Images alongside their resultant segmented results using distinct segmentation approaches, such as K-Means, Region Growing, BIRCH, Conventional Res U-Net and P-ResU-Net. Notably, the P-ResU-Net surpasses the established strategies, indicating exceptional segmentation effectiveness in identifying key features of lung cancer. Thereby, P-ResU-Net showcases its enhanced performance in lung cancer classification.

#### Analysis on dice, segmentation accuracy and jaccard

Table 6 explains the detailed segmentation performance of P-ResU-Net in terms of Dice, Segmentation Accuracy and Jaccard metrics. This evaluation compares the performance of P-ResU-Net with traditional strategies, such as Region Growing, K-Means, Birch, and Conventional Res U-Net. Examining the Dice measure, the P-ResU-Net accomplished the highest dice score of 0.929, surpassing traditional approaches like traditional Res U-Net (0.909), Birch (0.841), K-Means (0.843) and Region Growing (0.724), respectively. This signifies the excellent segmentation performance of P-ResU-Net in recognizing precise lung cancer regions. Similarly, the maximum segmentation accuracy acquired using the P-ResU-Net is 0.940, indicating its effectiveness in segmenting lung cancer regions. In contrast, the traditional strategies like Birch, K-Means, Region Growing and Conventional Res U-Net recorded lower segmentation accuracy ratings. The P-RDCAB improves the feature maps by highlighting important channels and suppressing less relevant ones. Also, it significantly improves the generalization ability of the P-ResU-Net.

**Table 6:** Segmentation Performance for P-ResU-Net Versus Existing Methods.

Measures	Region Growing	BIRCH	K-means	Traditional Res U-Net	P-ResU-Net
Dice	0.724	0.841	0.843	0.909	0.929
Segmentation Accuracy	0.776	0.853	0.863	0.910	0.940
Jaccard	0.709	0.800	0.854	0.899	0.917

### Comparative analysis

**Fig. 10:**
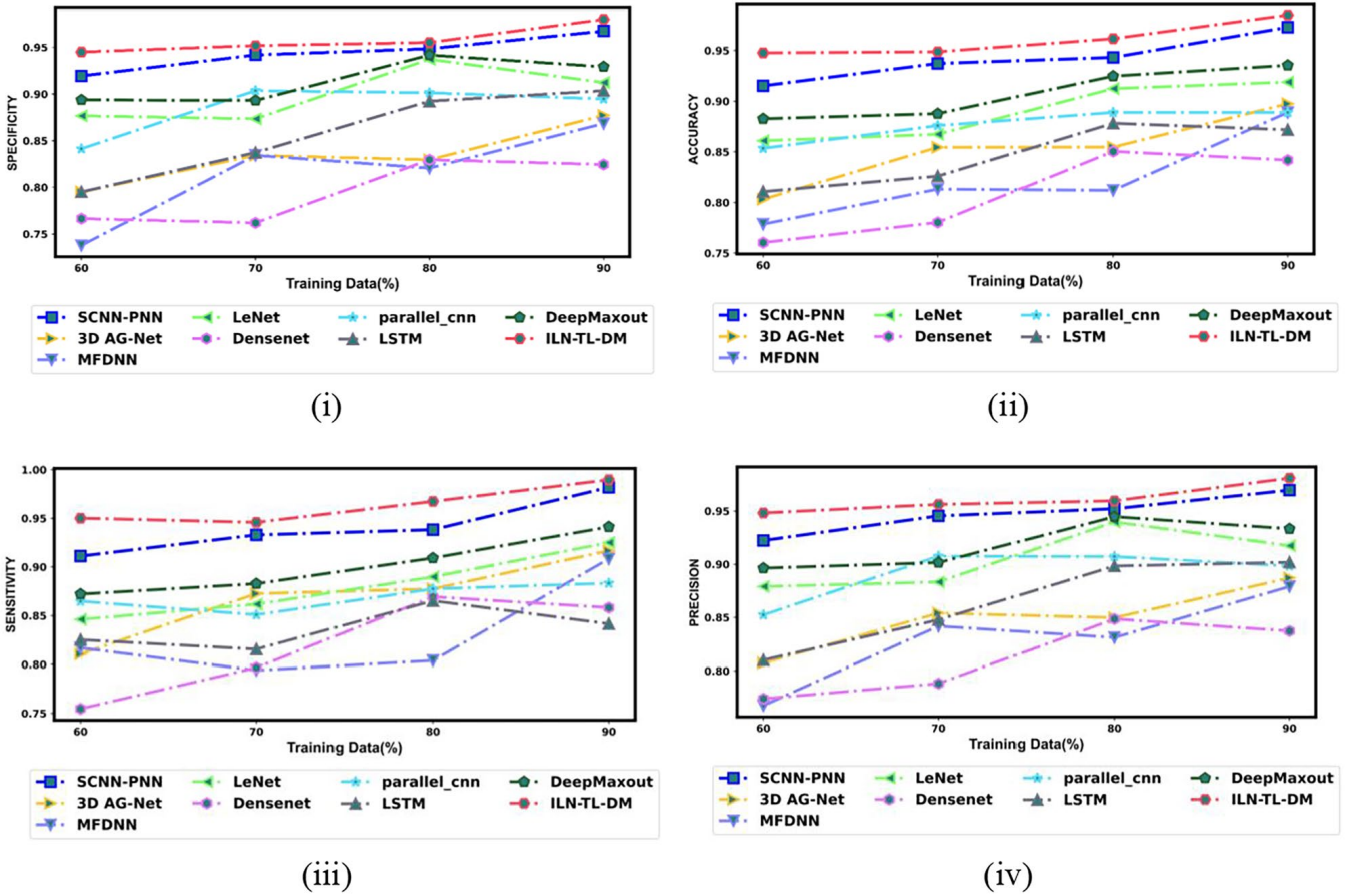
Assessment of Positive measures for ILN-TL-DM Vs. Traditional techniques (i) Specificity (ii) Accuracy (iii) Sensitivity and (iv) Precision.

**Fig. 11:**
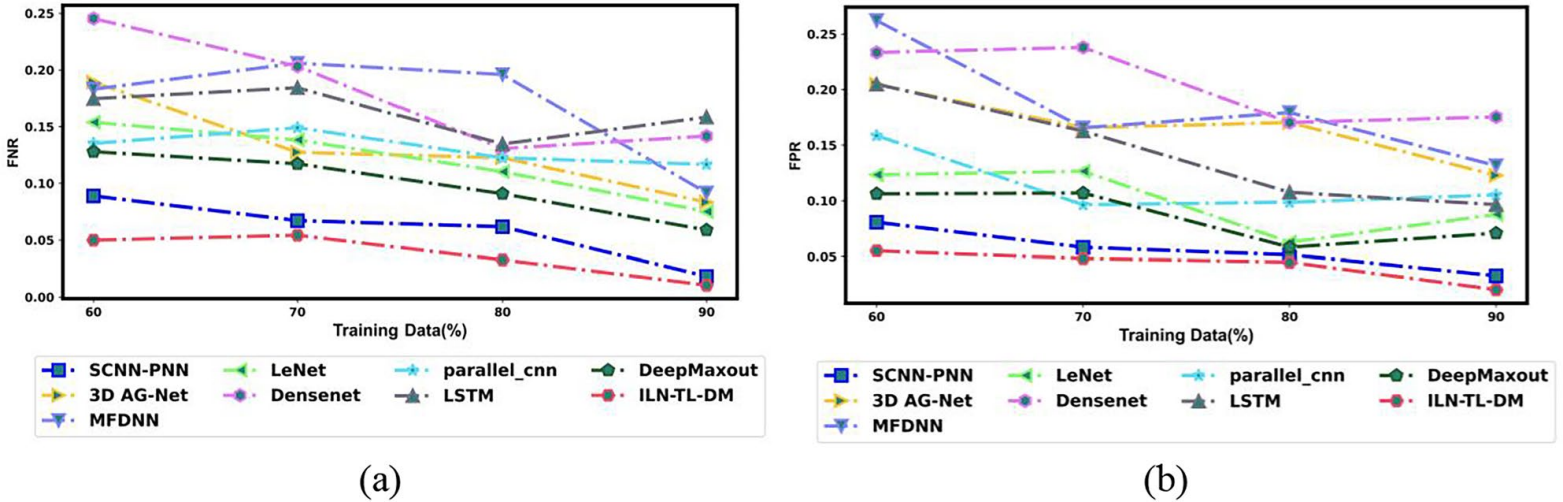
Assessment of Negative measures for ILN-TL-DM Vs. Traditional techniques (**a**) FNR and (**b**) FPR.

**Fig. 12:**
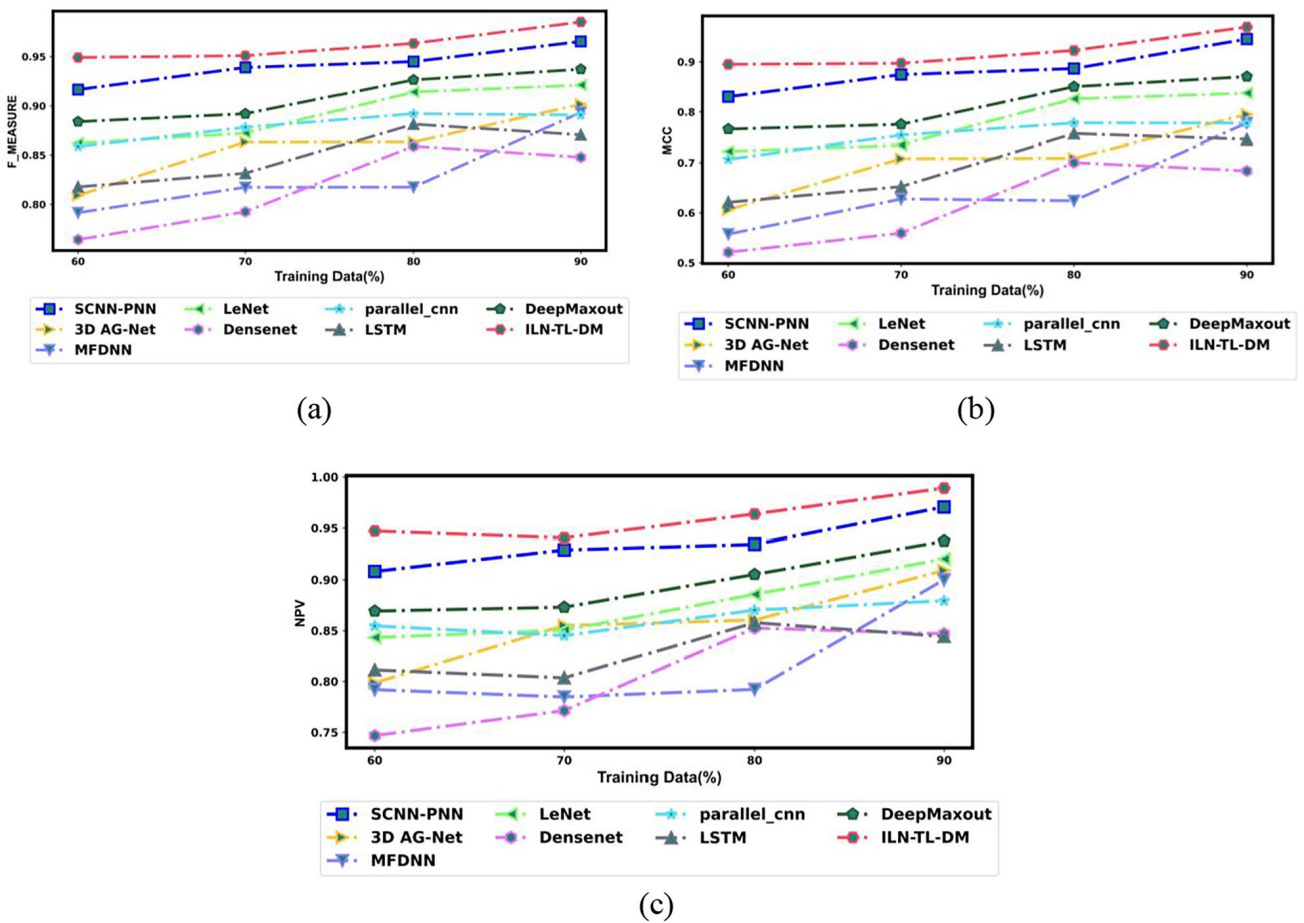
Assessment on other Metrics for ILN-TL-DM Vs. traditional techniques (**a**) F-measure (**b**) MCC and (**c**) NPV.

To validate the efficiency of the ILN-TL-DM approach for Lung Cancer Classification using CT images, a comparative assessment is made against conventional strategies like 3D AG-Net^26^, MFDNN^24^, SCNN-PNN^42^ LeNet, DenseNet, Parallel CNN, LSTM and Deep Maxout. The evaluation aimed to assess these approaches’ effectiveness using a range of indicators, such as positive, negative, and other measures. The results of this comparison analysis are shown in Figs. 10, 11, and 12. The model should be expected to perform well in both positive and other metrics in order to achieve an effective lung cancer classification, demonstrating its capacity to accurately classify lung cancer. Analyzing 80% training data, the ILN-TL-DM scheme accomplished the highest accuracy rate of 0.962, outperforming the conventional methods, such as 3D AG-Net^26^ at 0.855, MFDNN^24^ at 0.812, LeNet at 0.912, DenseNet at 0.850, Parallel CNN at 0.889, LSTM at 0.878, Deep Maxout at 0.925, and SCNN-PNN^42^ at 0.921, respectively. Regarding the Specificity measure, the ILN-TL-DM approach achieved the highest specificity of 0.945, outperforming the conventional approaches, which yielded lower specificity values of 0.767 to 0.894. The ILN-TL-DM continuously increased the specificity values to 0.952, 0.955, and 0.980 when the training data was improved to 70%, 80%, and 90%, respectively.

Additionally, the MCC metric provided excellent performance compared to traditional approaches across all training data. For 60% training data, the ILN-TL-DM scheme generated the MCC score of 0.895, significantly higher than 3D AG-Net^26^, MFDNN^24^, LeNet, DenseNet, Parallel CNN, LSTM, Deep Maxout and SCNN-PNN^42^. When the training data is augmented to 70%, the ILN-TL-DM model enhances the MCC to 0.897, and at 80% training data, it reaches 0.923. Finally, at 90% training data, the ILN-TL-DM achieved the peak MCC value of 0.970. In contrast, the conventional methods like 3D AG-Net^26^ (0.795), MFDNN^24^ (0.778), LeNet (0.837), DenseNet (0.684), Parallel CNN (0.778), LSTM (0.746), Deep Maxout (0.871), and SCNN-PNN^42^ (0.952) recorded relatively lower MCC values. Considering the Negative metric, which ought to be diminished for efficacious lung cancer classification. More particularly, the ILN-TL-DM scheme exhibited the least FPR score of 0.045 at 80% training, while the 3D AG-Net^26^, MFDNN^24^, LeNet, DenseNet, Parallel CNN, LSTM, Deep Maxout, and SCNN-PNN^42^ scored higher FPR ratings of 0.170, 0.179, 0.063, 0.170, 0.099, 0.108, 0.058, and 0.051 respectively. This reduction in error values demonstrates the robustness of the ILN-TL-DM approach in lung cancer classification. The Improved Entropy method introduces the dynamic modulation of the effects of both Shannon entropy and normalized entropy, based on the local characteristics of the segmented image. This modulation increases the model’s capacity to retain spatial distribution information, which is vital in correctly discriminating between the various regions within the segmented images.

### Analysis on ROC curve

**Fig. 13:**
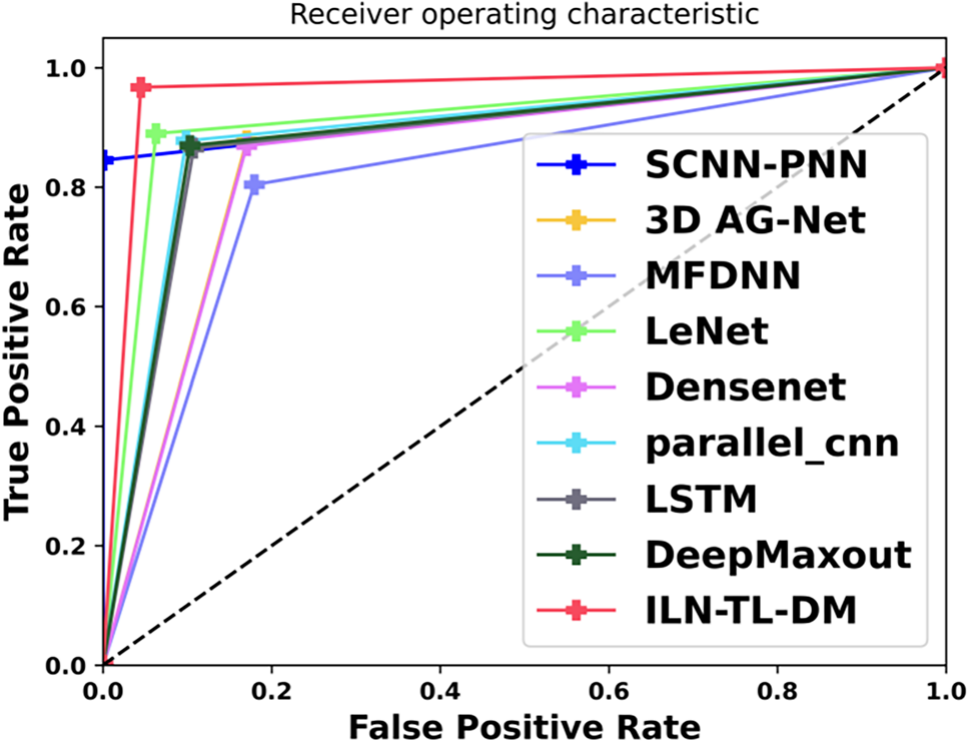
ROC Curve Estimation on ILN-TL-DM and Existing Strategies.

A ROC curve is a visual illustration of a classifier’s efficiency that contrasts the TPR and FPR across all feasible thresholds. Figure 13 exposes the ROC Curve evaluation of the ILN-TL-DM approach in comparison to traditional approaches, including 3D AG-Net^26^, MFDNN^24^, LeNet, DenseNet, Parallel CNN, LSTM, Deep Maxout, and SCNN-PNN^42^ for Lung Cancer Classification using CT images. This comparison highlights the ILN-TL-DM’s advantages over current methods in terms of classification accuracy and robustness, demonstrating its benefits in lung cancer detection. The ILN-TL-DM demonstrated exceptional efficacy in accurately identifying lung cancer by achieving an optimal AUC of 0.985. Comparatively, the traditional schemes like LeNet, Parallel CNN, Deep Maxout, DenseNet, 3D AG-Net^26^, LSTM and SCNN-PNN^42^ exhibit the AUC scores ranging between 0.827 to 0.876, representing excellent performance but not surpassing the ILN-TL-DM approach. The MFDNN^24^ established acceptable performance with an AUC score of 0.798. The ILN utilized the pretrained network, so the rich and hierarchical features can be easily learn by a deep pretrained network.

### Statistical analysis on accuracy

The detailed statistical evaluation on ILN-TL-DM for Lung Cancer Classification using CT images is illustrated in Table 7. This evaluation highlights the performance of the ILN-TL-DM scheme in comparison with traditional strategies, including 3D AG-Net^26^, MFDNN^24^, LeNet, DenseNet, Parallel CNN, LSTM, Deep Maxout, and SCNN-PNN^42^. In terms of Mean Statistical Measure, the ILN-TL-DM scheme exceeded all traditional methods by attaining the peak accuracy of 0.961. In contrast, the conventional methods like 3D AG-Net^26^, MFDNN^24^, LeNet, DenseNet, Parallel CNN, LSTM, Deep Maxout and SCNN-PNN^42^ demonstrated lesser accuracies. In addition, the ILN-TL-DM approach reached a higher accuracy of 0.985 under the Maximum statistical metric, showcasing its superiority in lung cancer categorization. In comparison, the LeNet and LSTM exhibited lesser accuracy of 0.850 and 0.878, while both MFDNN^24^ and Parallel CNN scored the accuracy of 0.889. The LeNet, Deep Maxout and SCNN-PNN^42^ obtained slight enhancements with accuracies of 0.919, 0.935, and 0.973, still below the ILN-TL-DM strategy. The ILN include more convolution layers and Gaussian normalization layers. This increased depth allows the network to learn a more hierarchical representation of data, potentially capturing more complex features, leading to better performance.

**Table 7:** Statistical Evaluation on Accuracy.

Metrics	SCNN-PNN ^42^ (%)	3D AG-Net ^26^ (%)	MFDNN 24 (%)	LeNet (%)	Densenet (%)	Parallel_cnn (%)	LSTM (%)	DeepMaxout (%)	ILN-TL (%)
Mean	94.20	85.20	82.30	89.00	80.80	87.70	84.70	90.80	96.10
Median	94.00	85.50	81.30	89.00	81.10	88.20	84.90	90.60	95.50
Std	2.10	3.30	4.00	2.60	3.90	1.40	2.90	2.30	1.50
Min	91.50	80.30	77.90	86.10	76.00	85.30	81.10	88.30	94.80
Max	97.30	89.70	88.90	91.90	85.00	88.90	87.80	93.50	98.50

### Ablation study

The ablation study analyzes the contribution of each component within the overall ILN-TL-DM framework, which emphasize the impact of each feature on overall classification performance. Table 8 describes the outcomes of an ablation assessment performed to analyze the effectiveness of the ILN-TL-DM Methodology for Lung Cancer Classification. This examination contrasts the ILN-TL-DM against several variants like ILN-TL-DM excluding Pre-processing, ILN-TL-DM with Existing Entropy, ILN-TL-DM with LGTrP feature only, ILN-TL-DM with PHOG feature only, ILN-TL-DM with Deep feature only and ILN-TL-DM with Existing Segmentation. The assessment is performed on 80% training data. The ILN-TL-DM obtained the minimum FNR rate of 0.033, underscoring its capability to accurately identify lung cancer. In comparison, the ILN-TL-DM, excluding pre-processing, acquired the higher FNR of 0.105, suggesting reduced categorization performance. Both the ILN-TL-DM with Existing Entropy and ILN-TL-DM with Deep feature only acquired the FNR values of 0.093. The ILN-TL-DM with LGTrP feature only, ILN-TL-DM with PHOG feature only and ILN-TL-DM with existing segmentation acquired the FNR values of 0.082, 0.097 and 0.077, respectively. This demonstrates the robustness of ILN-TL-DM in precisely categorizing lung cancer. The precision value attained using the ILN-TL-DM is 0.960, outperforming the ILN-TL-DM without Pre-processing, ILN-TL-DM with Existing Entropy, ILN-TL-DM with LGTrP feature only, ILN-TL-DM with PHOG feature only, ILN-TL-DM with Deep feature only and ILN-TL-DM with Existing Segmentation generated the least precision ratings.

**Table 8:** Ablation study on ILN-TL-DM model, ILN-TL-DM without Pre-processing, ILN-TL-DM with Existing Entropy, ILN-TL-DM with LGTrP feature only, ILN-TL-DM with PHOG feature only, ILN-TL-DM with Deep feature only and ILN-TL-DM with Existing Segmentation.

Metrics	ILN-TL-DM without Pre-processing	ILN-TL-DM with Existing Entropy	ILN-TL-DM with LGTrP feature only	ILN-TL-DM with PHOG feature only	ILN-TL-DM with Deep feature only	ILN-TL-DM with Existing Segmentation	ILN-TL-DM
Precision	0.881	0.904	0.915	0.885	0.904	0.920	0.960
FPR	0.124	0.102	0.090	0.120	0.102	0.085	0.045
Sensitivity	0.895	0.907	0.918	0.903	0.907	0.923	0.967
FNR	0.105	0.093	0.082	0.097	0.093	0.077	0.033
MCC	0.850	0.811	0.832	0.862	0.811	0.843	0.923
Specificity	0.876	0.898	0.910	0.880	0.898	0.915	0.955
NPV	0.892	0.901	0.913	0.900	0.901	0.918	0.964
F-measure	0.888	0.905	0.916	0.893	0.905	0.922	0.963
Accuracy	0.886	0.902	0.914	0.892	0.902	0.919	0.962

Table 9 presents an ablation study comparing the performance of a model incorporating different entropy-based feature extraction techniques. Specifically, the table contrasts models using Renyi entropy, Deng entropy, Normalized entropy, Conventional Shannon entropy, and the proposed Improved entropy, which is designed as a weighted sum of Shannon entropy and Normalized entropy. Several evaluation criteria, including specificity, precision, F-measure, MCC, NPV, FPR, accuracy, sensitivity, and FNR, form the basis of the comparison. With the best precision (0.960), F-measure (0.963), accuracy (0.962), and MCC (0.923), the model that uses improved entropy demonstrates a greater capacity to differentiate between cancerous and non-cancerous tissue. In contrast, models using Renyi, Deng, Normalized, and conventional Shannon entropy show relatively lower performance, specifically, the model with the conventional Shannon entropy performing the worst overall. This ablation study strongly supports the integration of the Improved entropy method within the feature extraction pipeline of the proposed ILN-TL-DM architecture. Incorporating the weight factor in the improved entropy significantly enhances classification performance across all metrics, demonstrating its utility in improving diagnostic accuracy for the classification of lung cancer from CT images.

**Table 9:** Ablation analysis on the model with improved entropy Vs the model with conventional entropy methods.

Metrics	Model with Renyi entropy (%)	Model with deng entropy (%)	Model with normalized entropy (%)	Model with traditional shannon entropy (%)	Model with Improved entropy (%)
Accuracy	93.00	92.50	93.90	90.20	96.20
Sensitivity	93.40	92.90	94.40	90.70	96.70
Specificity	92.60	92.00	93.40	89.80	95.50
Precision	92.90	92.30	93.70	90.40	96.00
F-measure	93.20	92.60	94.00	90.50	96.30
MCC	88.30	87.30	89.30	81.10	92.30
NPV	93.20	92.60	94.10	90.10	96.40
FPR	7.40	8.00	6.60	10.20	4.50
FNR	6.60	7.10	5.60	9.30	3.30

### Analysis on computational time

The term “computational time” describes how long it takes a model to finish a certain task. The computation time analysis of the suggested ILN-TL-DM model is presented in Table 10 along with comparisons to the current approaches, including LeNet, Densenet, SCNN-PNN, 3D AG-Net, MFDNN, Parallel_CNN, LSTM, and DeepMaxout. The results show that the suggested ILN-TL model is the most efficient, achieving the shortest computing time (30.416 s) out of all the models that were evaluated. On the contrary, the LeNet, DeepMaxout, 3D AG-Net, and Densenet perform with relatively low times of 32.382 s, 38.364 s, 82.303 s and 74.000 s, respectively. Additionally, models like SCNN-PNN, MFDNN, Parallel_CNN and LSTM achieve minimal computing time of 61.260 s, 48.176 s, 55.394 s and 54.789 s. Overall, the analysis shows that ILN-TL and DeepMaxout, which are essential parts of the suggested hybrid ILN-TL-DM model, preserve computational efficiency and improve classification accuracy, making it appropriate for real-time lung cancer detection.

**Table 10:** Assessment of computational time.

Models	Computing Time(sec)
SCNN-PNN	61.260
3D AG-Net	82.303
MFDNN	48.176
LeNet	32.382
Densenet	74.000
Parallel_CNN	55.394
LSTM	54.789
DeepMaxout	38.364
ILN-TL-DM	30.416

### Runtime analysis

Table 11 presents the runtime comparison of the proposed ILN-TL-DM model against several versions of the model. The runtime refers to the total duration (in seconds) required for the complete execution of the model to the final classification output. The proposed ILN-TL-DM model recorded a runtime of 3132.29 s, which, while not the lowest, represents a balanced trade-off between speed and accuracy, meanwhile the runtime of the model without segmentation is 2654.216 s, model omitting preprocessing is 3006.339 s, models using conventional entropy is 4123.625 s and model using traditional ResU-Net segmentation is 4024.565 s. The key innovation lies in the integration of adaptive preprocessing, improved Attention-based ResU-Net segmentation, and the extraction of improved entropy features, along with the integration of ILN-TL and DeepMaxout models are optimized to maintain accuracy without excessive runtime. This indicates that the improvements integrated into the ILN-TL-DM model reduce overall runtime while maintaining high classification accuracy of lung cancer, making it a practical choice for clinical applications where both speed and precision are critical.

**Table 11:** Assessment of runtime on the proposed ILN-TL-DM model Vs the Model without preprocessing, Model using conventional Entropy, Model with conventional ResU-Net segmentation and Model without segmentation.

Models	Running Time(sec)
Model without preprocessing	3006.339
Model using conventional Entropy	4123.625
Model with conventional ResU-Net segmentation	4024.565
Model without segmentation	2654.216
ILN-TL-DM	3132.29

### Analysis on confusion matrix

**Fig. 14:**
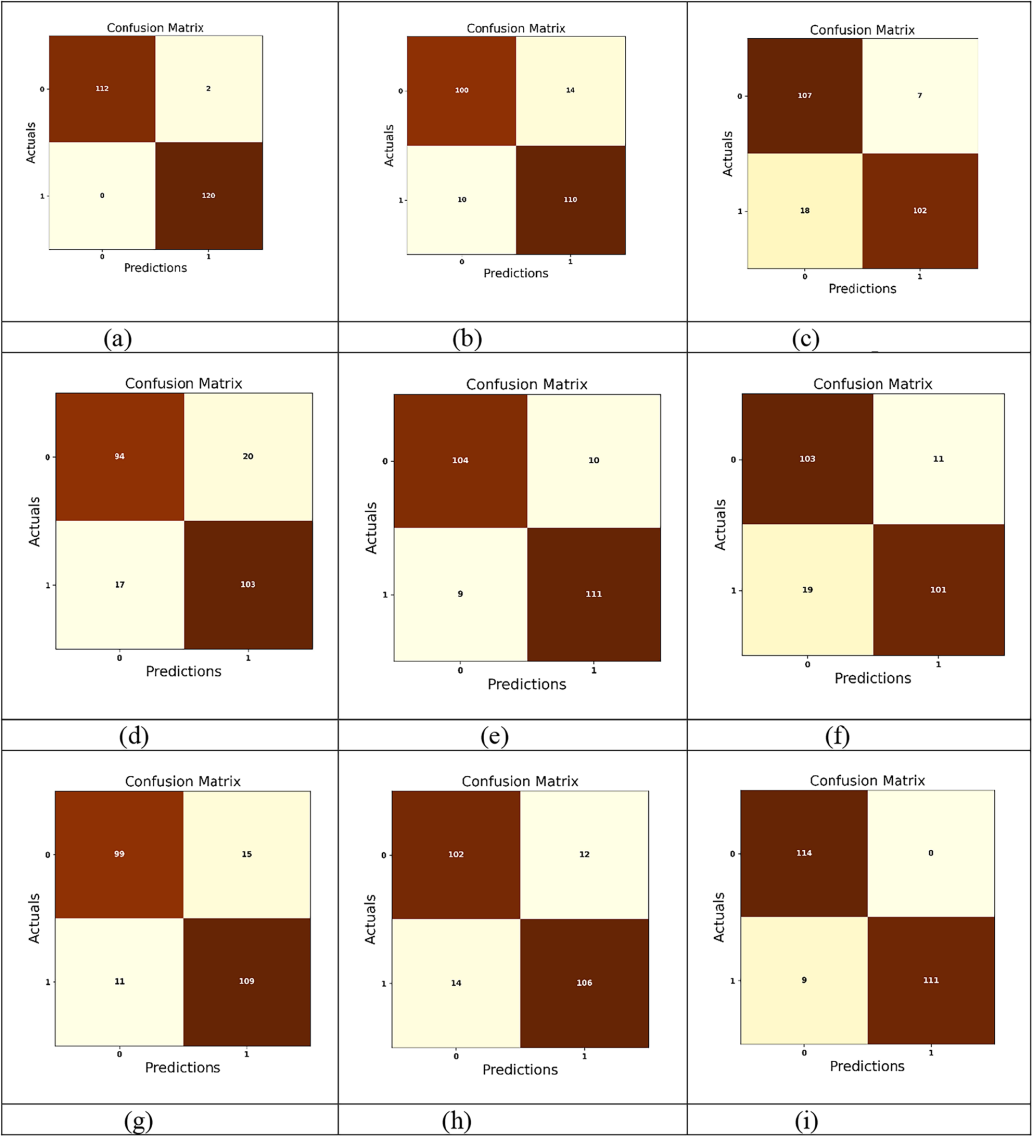
Confusion matrix analysis on (**a**) ILN-TL-DM (**b**) 3D AG-Net (**c**) DeepMaxout (**d**) DenseNet (**e**) LeNet (**f**) LSTM (**g**) MFDNN (**h**) Parallel-CNN and (**i**) SCNN-PNN.

An effective technique for assessing how well classification algorithms perform is a confusion matrix, sometimes referred to as an error matrix. A straightforward evaluation of a model’s classification behavior is made possible by the matrix’s rows, which reflect the actual classes, and columns, which show the predicted classes. Key evaluation metrics, such as TPR, TNR, FPR, and FNR, are derived from this matrix to quantify model effectiveness. Figure 14 presents the confusion matrix comparison between the suggested ILN-TL-DM method and several conventional models, including SCNN-PNN, 3D AG-Net, MFDNN, LeNet, Densenet, Parallel_CNN, LSTM, and DeepMaxout. The results of this study, which concentrates on binary classification, absolutely show that the ILN-TL-DM model performs better than other models, especially when it comes to correctly identifying the genuine class labels. With a low FP rate of 2 and a noticeably larger TP rate of 112, the suggested model shows a markedly lower misclassification rate. These results demonstrate how well the ILN-TL-DM model classifies lung cancer from CT images in terms of accuracy and dependability.

## Conclusion

In summary, this study effectively proposed a new hybrid ILN-TL-DM model for lung cancer classification from CT images. The method effectively solved the issues of noise elimination, precise segmentation, and feature extraction using sophisticated methods like Adaptive Gaussian filtering, Improved Attention-based ResNet, and improved entropy-based features. Compared to traditional methods, the combination of a DeepMaxout classifier and an improved LeNet model incorporating Transfer Learning and soft voting performed better in lung cancer classification. The proposed method offers enhanced precision, resilience, and generalization, making it a viable option for the early identification of lung cancer in medical imaging. The results demonstrated that applying hybrid deep learning approaches could improve the precision and reliability of cancer detection techniques and lead to the development of more advanced diagnostic medical instruments. Analyzing 80% training data, the ILN-TL-DM scheme accomplished the highest accuracy rate of 0.962, outperforming the conventional methods, such as 3D AG-Net^26^ at 0.855, MFDNN^24^ at 0.812, LeNet at 0.912, DenseNet at 0.850, Parallel CNN at 0.889, LSTM at 0.878 and Deep Maxout at 0.925, respectively. While P-ResU-Net is an advanced segmentation model, lung CT images can be highly heterogeneous due to variations in tumor shape, size, position, and surrounding tissue structures. The segmentation model might still fail in some edge cases, such as when the tumor is too small or poorly defined. Incorrect segmentation of tumor regions can lead to poor feature extraction and impact on the overall classification performance.

## Data Availability

The datasets generated and/or analyzed during the current study are available online and are publicly accessible at:https://www.kaggle.com/datasets/avc0706/luna16/data?select=seg-lungs-LUNA16.
